# Mapping Ethanol Tolerance in Budding Yeast Reveals High Genetic Variation in a Wild Isolate

**DOI:** 10.3389/fgene.2019.00998

**Published:** 2019-11-20

**Authors:** Roni Haas, Guy Horev, Ehud Lipkin, Inbar Kesten, Maya Portnoy, Keren Buhnik-Rosenblau, Morris Soller, Yechezkel Kashi

**Affiliations:** ^1^Faculty of Biotechnology and Food Engineering, Technion, Haifa, Israel; ^2^Lorey I. Lokey Interdisciplinary Center for Life Sciences and Engineering, Technion–Israel Institute of Technology, Haifa, Israel; ^3^Department of Genetics, Silberman Life Sciences Institute, The Hebrew University of Edmond Safra Campus, Jerusalem, Israel

**Keywords:** QTL mapping, allele effect, genetic architecture of complex trait, causative genes, Saccharomyces cerevisiae

## Abstract

Ethanol tolerance, a polygenic trait of the yeast *Saccharomyces cerevisiae*, is the primary factor determining industrial bioethanol productivity. Until now, genomic elements affecting ethanol tolerance have been mapped only at low resolution, hindering their identification. Here, we explore the genetic architecture of ethanol tolerance, in the F6 generation of an Advanced Intercrossed Line (AIL) mapping population between two phylogenetically distinct, but phenotypically similar, *S. cerevisiae* strains (a common laboratory strain and a wild strain isolated from nature). Under ethanol stress, 51 quantitative trait loci (QTLs) affecting growth and 96 QTLs affecting survival, most of them novel, were identified, with high resolution, in some cases to single genes, using a High-Resolution Mapping Package of methodologies that provided high power and high resolution. We confirmed our results experimentally by showing the effects of the novel mapped genes: *MOG1*, *MGS1*, and YJR154W. The mapped QTLs explained 34% of phenotypic variation for growth and 72% for survival. High statistical power provided by our analysis allowed detection of many loci with small, but mappable effects, uncovering a novel “quasi-infinitesimal” genetic architecture. These results are striking demonstration of tremendous amounts of hidden genetic variation exposed in crosses between phylogenetically separated strains with similar phenotypes; as opposed to the more common design where strains with distinct phenotypes are crossed. Our findings suggest that ethanol tolerance is under natural evolutionary fitness-selection for an optimum phenotype that would tend to eliminate alleles of large effect. The study provides a platform for development of superior ethanol-tolerant strains using genome editing or selection.

## Introduction

The yeast *Saccharomyces cerevisiae* has long been studied as a model organism for eukaryotic molecular and cellular biology. S. cerevisiae’s genome was the first eukaryote genome completely sequenced, with haploid genome of 12,068 kb and 5,885 potential protein encoding genes in 16 chromosomes (Goffeau et al., 1996).

Ethanol, the most consumed biofuel worldwide, is the main fermentation end product of *S. cerevisiae*. Yeast naturally produces ethanol during anaerobic fermentation of sugar. Ethanol toxicity may inhibit cell division and viability, reduce metabolic activity, harm cell transport and change composition, structure, and function of cellular membranes, proteins, and morphology (van Voorst et al., 2006; Ding et al., 2009; Liu and Qureshi, 2009; Avrahami-Moyal et al., 2012). High ethanol concentration endangers the survival of the cells, while under moderate ethanol levels, cells survive but may have very reduced growing ability and fermentation rate. Thus, both aspects of ethanol tolerance, growth, and survival are key factors in ethanol production (Amorim et al., 2011). Hence, uncovering the genetic basis and molecular mechanisms of ethanol tolerance in yeast is necessary to improve the efficiency of biofuel producing strains. However, ethanol tolerance of yeast is a polygenic and complex quantitative trait, and analysis of such traits is a major challenge in genetics (Swinnen et al., 2012b). One of the greatest challenges is the identification of minor quantitative trait loci (QTLs), while QTLs of large effect are more easily found (Brem et al., 2005; Demogines et al., 2008; Sinha et al., 2008; Ehrenreich et al., 2010; Parts et al., 2011; Swinnen et al., 2012b). A previous ethanol tolerance mapping study by Hu et al., 2007, based on the F2 of a cross between two strains that diverged widely in ethanol tolerance, uncovered two QTLs of large and three QTLs of moderate effect on ethanol tolerance, as measured by survival. Another study, of similar design, in which a large-scale QTL mapping experiment was carried out, detected one QTL of large effect for ethanol tolerance as measured by growth (Cubillos et al., 2011).

In the last decade, *S. cerevisiae* has become a powerful model organism for quantitative genetics, thanks to recently developed technologies to cross strains and generate large numbers of segregants (Bahalul et al., 2010; Liti and Louis, 2012; Fay, 2013). Recently, genome-wide QTL mapping by selective DNA pooling, (SDP, also termed “Bulk Segregant Analysis”, BSA), was carried out in yeast. For purposes of estimating population allele frequencies and for QTL mapping, genotyping pools is much more cost effective than genotyping individuals and allows much larger samples. Larger samples result in smaller sampling variance and therefore improve the accuracy of allele frequency estimates (Schlotterer et al., 2014) and increase power to reveal loci with small effects (Korol et al., 2007; Ehrenreich et al., 2010; Duitama et al., 2014). Previous yeast studies used BSA of F2 segregants with sequencing of DNA pools, to identify novel genes related to ethanol tolerance (Swinnen et al., 2012a; Pais et al., 2013; Wilkening et al., 2014). Several QTL spanning more than one gene were found, and further investigations specifically identified six genes related to the trait.

The Advanced Intercrossed Line (AIL) design is based on crossing two or more genetically distinct pure lines, followed by several generations of intercrossing (Darvasi and Soller, 1995). Recombinations accumulated over the intercross generations produce mosaic genomes composed of a mix of very small segments of founder genomes. This breaks up long-distance linkage disequilibrium (LD); possibly also breaking up balanced linkage blocks, keeping appreciable LD only between very close sites (**Supplementary Figure 1**), thus increasing mapping resolution (Darvasi and Soller, 1995). Parts et al., 2011, using a yeast two-parent AIL, were able to map 21 heat tolerance QTLs. This was far more than the number of QTLs ordinarily obtained by an F2 linkage analysis. A four-parent AIL design with the potential for four participating alleles (Cubillos et al., 2013), mapped 34–39 heat tolerance QTLs.

QTL mapping for ethanol tolerance has generally involved industrial strains selected to differ widely in this trait (Swinnen et al., 2012a; Pais et al., 2013). However, wild populations of many domesticated plants are rich sources of genetic variants affecting a wide variety of quantitative traits of agro-economic importance (Luikart et al., 2012). One of the aims of the present study was to examine this in yeast. To this end, we implemented genome-wide SDP mapping for QTL affecting ethanol tolerance in an AIL population based on a cross between the standard haploid laboratory strain S288c, and YE-531, a haploid strain isolated from nature (**Supplementary Figure 1**). The two strains belong to different lineages but are of similar phenotype for ethanol tolerance. Genome-wide AIL QTL mapping has not been previously conducted for ethanol tolerance. We studied growth and survival in the presence of alcohol as two distinct traits, as both are important for commercial purposes. By applying a multi-component package of experimental and statistical procedures, numerous QTLs were identified, spanning narrower regions than previously reported by non-AIL studies; in some cases down to single genes. The parental strains, although of similar tolerance phenotype, were found to contain a very large amount of genetic variation in the form of numerous balanced QTLs of small but mappable effect.

## Results

### Phylogenetically Distinct Parental Strains With Similar Phenotypic Performance

An AIL population was produced by crossing a haploid laboratory strain, S288c, of known genome sequence, with a heterothallic haploid strain, YE-531, isolated from nature (Ezov et al., 2006). We choose a standard laboratory strain, and a strain from the wild, to explore usefulness of wild yeast as source of positive alleles for traits of economic or biological importance. The two haploid strains had similar mean phenotypic values (**Supplementary Figure 2**). Population sizes were >10^5^ during the intercross phase. During the interval between sexual generations, there would have been opportunity for one to three generations of vegetative reproduction. Consequently, some differential clonal reproduction may have occurred in the intervals between sexual generations.

**Figure 1:**
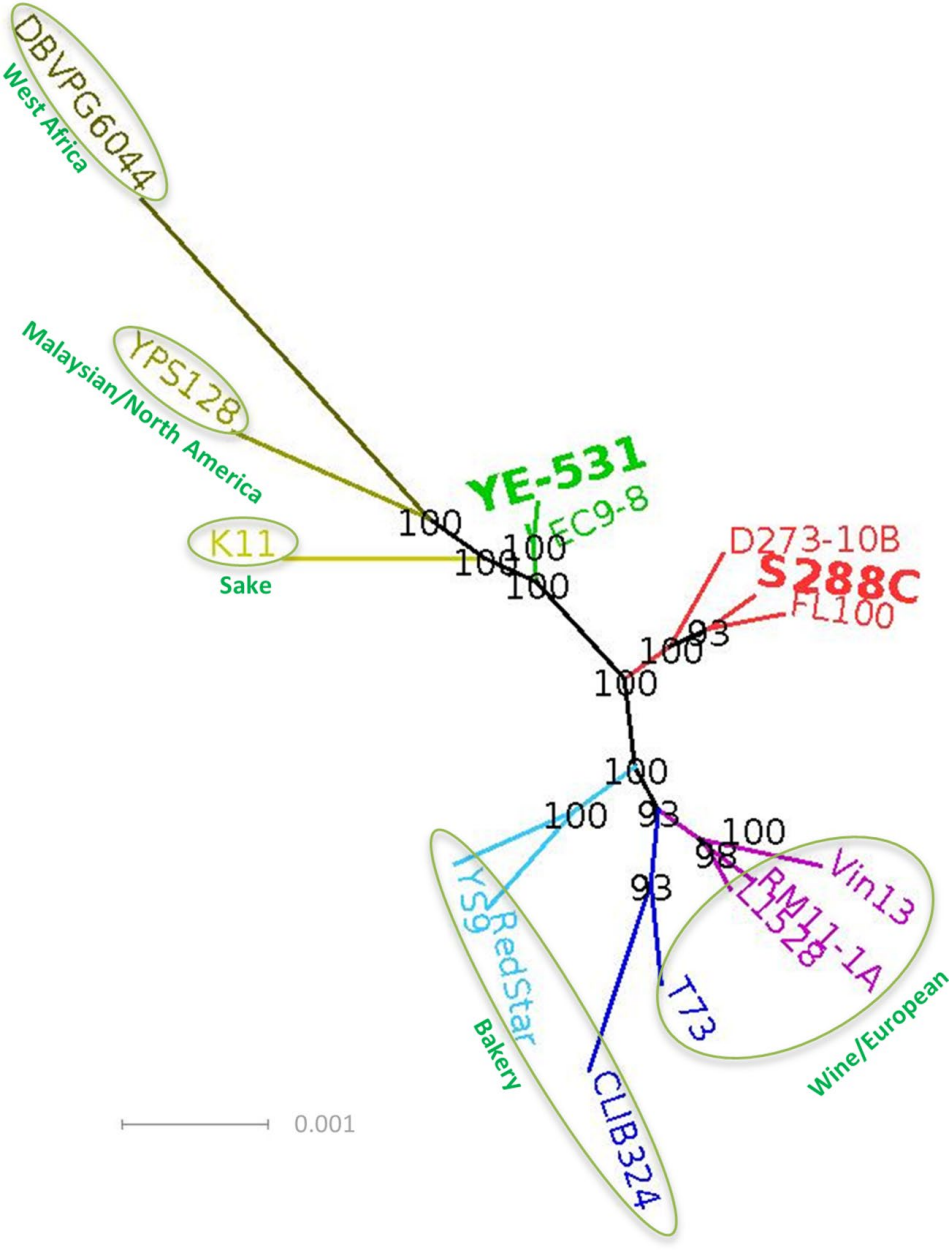
Phylogenetic analysis, based on whole genome sequences, of the AIL parental lines in relation to known *S. cerevisiae* strains representing different lineages and groups. The genome of YE-531 was generated by *de novo* sequencing. Colored text, genome name of the strain; green circle, lineages (Liti et al., 2009; Schacherer et al., 2009; Maclean et al., 2017); Bolded and enlarged, AIL parental lines. Bootstrap values are presented on the branches in black. The branch length units are the number of changes in the sequence divided by the sequence length in bp. The full sequence identifiers of the genome sequences we used for the tree building and the strains description are summarized in **Supplementary Table 1**.

We obtained the genome of YE-531 by *de novo* sequencing. Then we used the full genomes of the two founders and 13 publicly available *S. cerevisiae* genomes of different lineages (Liti et al., 2009; Schacherer et al., 2009; Maclean et al., 2017) to determine phylogenetic relationships. **Figure 1** shows clearly that YE-531 is distinct from S288c, as well as from the other wild and commercial yeast strains, while one wild strain, EC9-8a, is phylogenetically close to YE-531. This is reassuring, as both strains were isolated from “Evolution Canyon”, Mt. Carmel, Israel (Ezov et al., 2006; Chang and Leu, 2011).

### Forming the Pools for SDP Mapping

**Figure 2:**
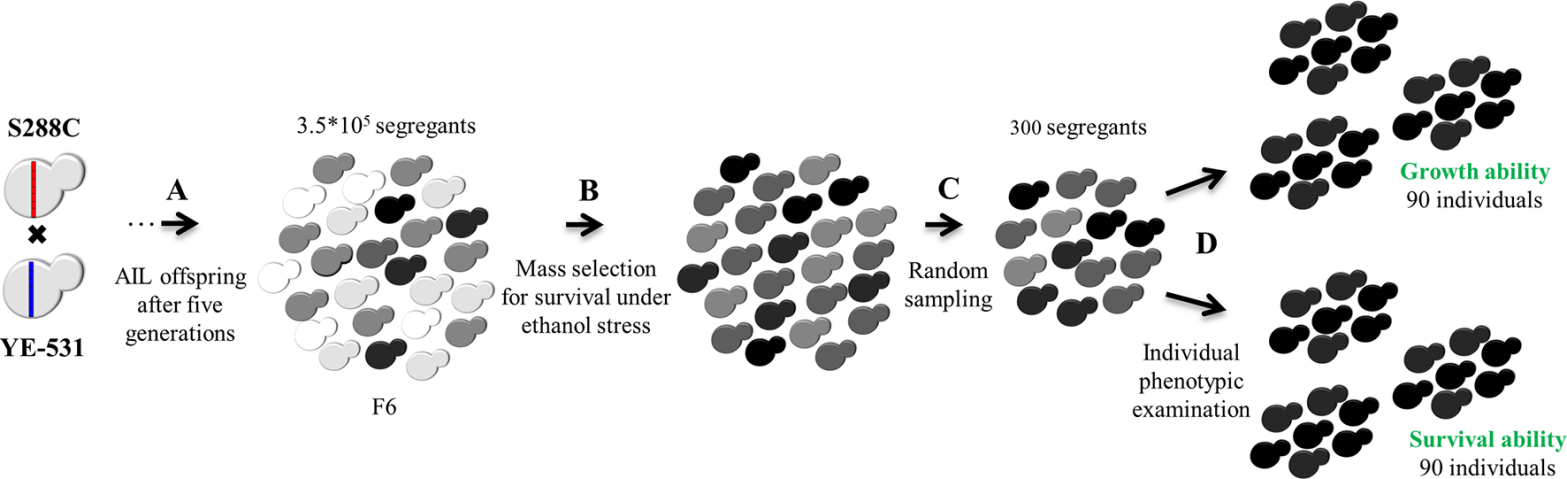
AIL and tail selection. **(A)** F1 offspring were intercrossed for five generations to create F6. **(B)** First stage: Mass-selection for survival under moderate (15% V/V) ethanol stress (35% survivors) to create initial phenotypic upper tail group common to both traits. **(C)** Random sampling of 300 segregants from the initial upper tail group. **(D)** Individual phenotypic evaluation of these 300 segregants separately for growth and for survival, under ethanol stress, to construct two final selected pools containing the highest 90 segregants each for growth and survival in the presence of ethanol. The entire F6 population was used as a control for both traits. For statistical purposes, each pool and control group was randomly divided into three subgroups. Cell colors represent variation in ethanol tolerance in the population. Darker cells indicate higher ethanol tolerance.

For purpose of QTL mapping by SDP, a two-stage selection scheme was used as shown in **Figure 2**, to construct the ethanol-tolerant selected tail pools for growth and survival. In the first stage, a level of ethanol stress that was survived by 35% of the population was used to select ethanol tolerant colonies. This stage was common to both traits. In the second stage, 300 colonies were tested separately for growth and for survival, and 30% (90 colonies out of 300) were chosen for each trait. This retains essentially all of the mapping information of the entire mapping population of 300 individual colonies (Darvasi and Soller, 1992). Unselected aliquots from the AIL-F6 population served as controls. To determine marker allele frequencies in the pools and controls we performed whole genome sequencing.

### Allele Frequency Variation in F6 Is Higher Than Expected

Although the markers in the F1 are expected to have allele frequency 0.5, some variation in marker frequency is anticipated in the F6 due to sampling. Surprisingly, there was very wide variation in marker allele frequencies in the F6 population (**Supplementary Table 2**).

Given the very large population sizes used in producing the successive generations, the observed variation in marker allele frequencies at the F6 generation is considerably larger than would be expected by cumulative binomial sampling. Even for effective population size of only 1,000, binomial sampling would generate a frequency standard deviation of no more than 0.02 per generation, or 0.06 across six generations. Thus, the remainder may have been generated by clonal reproduction and general fitness selection during the recuperation stages of the six AIL generations. As noted, S288c and YE-531 represent different lineages (**Figure 1**). Thus, considerable selection is expected as the two genomes adjust to the mutually novel genetic background generated by the cross. A further selection may be imposed by the Ether–zymolyase ascospore isolation procedure (see *Materials and Methods*) (Bahalul et al., 2010). The selective forces, whatever their nature, appear to have affected alleles originating from both parental lines more or less equally. In the F6 general population, the major allele originated from YE-531 in 47.2% of markers, and in the remaining 52.8% from S288c (**Supplementary Table 2**). The small excess of S288c alleles may be due to greater adaptation of the laboratory strain S288c to laboratory conditions. Interestingly, there were no single nucleotide polymorphism (SNPs) at which the YE-531 allele was lost or fixed in the F6.

### QTL Mapping by SDP

Following frequency smoothing for location by locally weighted scatterplot smoothing (LOESS) (Chambers and Hastie, 1992), mapping QTLs by means of SDP was based on the difference (D-value) of YE-531 marker allele frequency between the selected and control groups. We note that LOESS does not induce dependency between independent markers; rather, it removes noise, so it will improve correlation estimates between linked markers. Comparison of the two traits reveals some regional overlaps of the D-values, along with regions where D-values of the two traits differ (**Supplementary Figure 3**).

The 90 individuals selected in each growth or survival pool were randomly assigned to three independent replicate subpools of 30 diploid individuals (60 chromosomes) each. Averaged across all markers, the variance estimates after LOESS were 0.0010, 0.0034, and 0.0044 among replicates of the control, growth, and survival pools, respectively. These observed values are plausible (**Supplementary text**) and are based on many thousands of data points each. Based on these estimates, empirical average standard errors of the differences between selected and control pools were 0.038 and 0.042 for growth and survival, respectively.

Using D-values of individual markers after LOESS smoothing for location (**Supplementary Table 3**), and the empirical SE adjusted for allele frequency, Comparison-Wise Error Rate (CWER) P-values were obtained for each of the 35,000+ markers for each trait (**Supplementary Data Sheet 1**, **2**). **Supplementary Table 4** shows the distribution of P-values for growth and survival. For both traits there was a large excess of P-values in the lowest P-value bin (P ≤ 0.10), compared to the proportion of 0.10 expected for this and all bins under the null hypothesis. This indicates the presence of a large number of rejected null hypotheses, i.e., of true marker–QTL associations. Based on the iterative procedure of Mosig et al. (2001), we estimated the number of true null hypotheses as n_2_ = 26,278 and 22,912 for growth and survival, respectively. Then, subtracting n_2_ from the total number of markers gave the estimates of rejected null hypothesis, n_1_, as 8,856 and 12,107 for growth and survival, respectively (**Table 1**). This is also illustrated by the Schweder-Spjotvoll plot (Spjotvoll, 1982) (**Supplementary Figure 4**; **Supplementary text**). Thus, it is estimated that 25% (8,856/35,134) and 35% (12,107/35,019) of the markers are linked to a causative mutation affecting growth or survival under ethanol stress, respectively.

**Table 1:** Critical CWER P-value thresholds for declaring marker significance, number of declared significant markers, and power according to FDR^1^.

FDR	Growth			Survival		
	Critical P	Significant markers	Power^3^	Critical P	Significant markers	Power^3^
≤0.001	2.0E-05	37	0.004	4.8E-05	19	0.002
≤0.010	3.4E-04	94	0.011	8.2E-04	547	0.045
≤0.050	3.3E-03	383	0.041	6.4E-03	1,875	0.147
≤0.100	9.6E-03	1,650	0.168	1.6E-02	3,700	0.275
≤0.200	3.4E-02	3,043	0.275	4.6E-02	7,498	0.495
n_1_	–	8,856	–	–	12,107	–
n_2_	–	26,278	–	–	22,912	–
Total	–	35,134	–	–	35,019	–

^1^In calculating power, the numbers of significant markers were reduced by 20% to take FDR into account.

^2^n_1_, estimated number of rejected null hypothesis; n_2_, estimated number of true null hypotheses.

^3^The power is (the number of markers significant in this P-value range after accounting for false discoveries)/n_1_.

Based on these n_2_ estimates, the power of the test (Mosig et al., 2001) and the number of markers reaching significance levels according to trait for various levels of FDR, are presented in **Table 1**. To declare significance we chose an FDR threshold of 0.2. Although this eliminates many markers that have high likelihood of representing true effects (see Power according to FDR in **Table 1**), it does not result in a corresponding loss of declared QTLs. This is because most of the excluded markers represent singleton tests that reach high significance by technical error of one sort or other; or represent additional markers associated with the same QTLs, that reached slightly lower D-values than the top markers in the QTL due to sampling or distance from the causative mutation.

### Mapping of Multiple Genetic Loci With High Resolution

**Figure 3:**
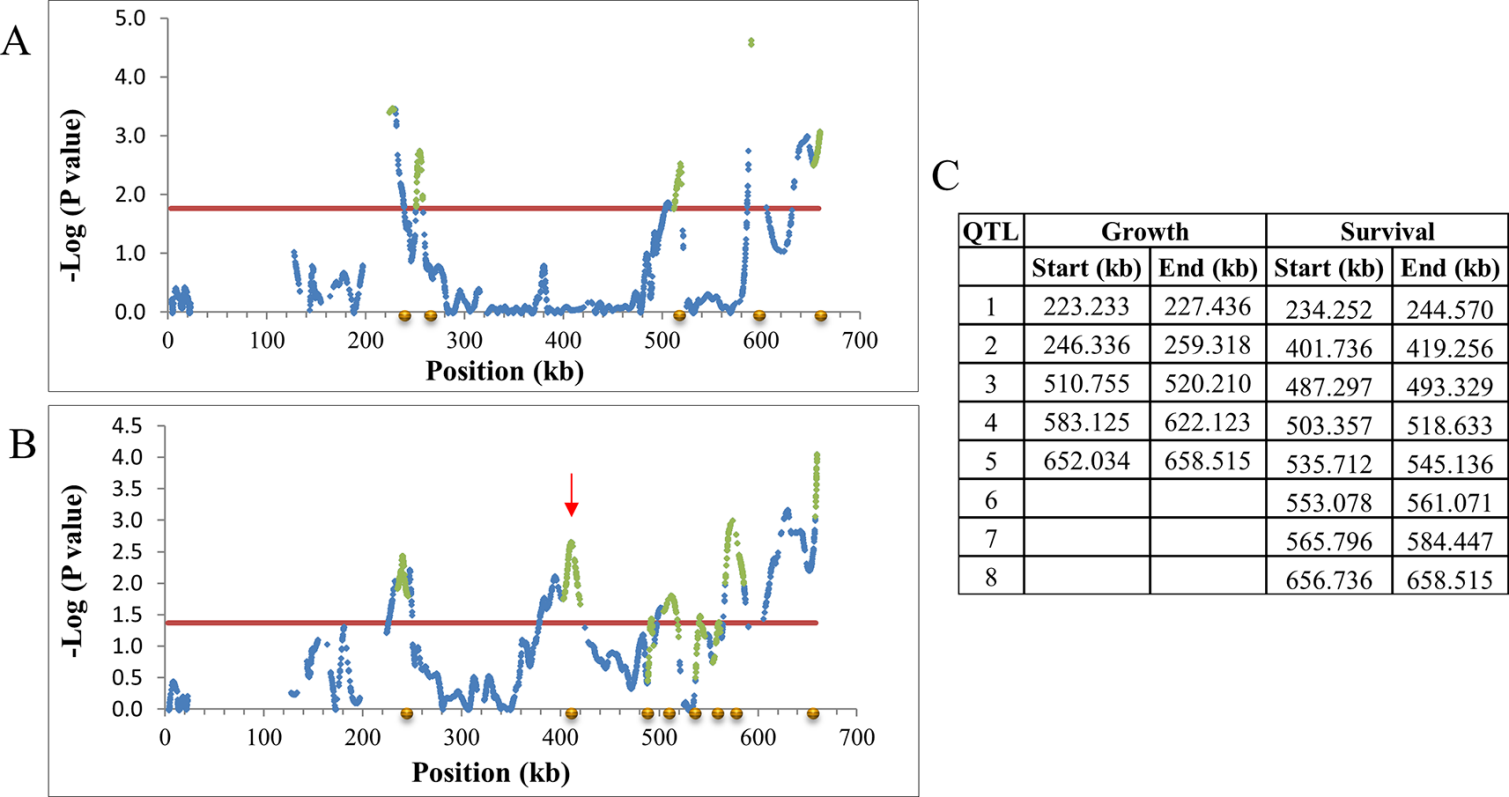
Declaring QTLs on chromosome 11. -Log_10_P values of SNPs after LOESS smoothing of allele frequency by location and SD adjustment by allele frequency were plotted against the marker position. Significant regions are those above FDR 0.2 and after log-drop procedure as in Lipkin et al., 2016. Blue diamond, smoothed SNP -log P-value; red line, 0.2 FDR; Green diamond, QTL after log-drop procedure. **(A)** Growth. The position of five declared QTLs is marked by gold circles on the X axis. the gap around location 210 is a result of lack of SNPs at that region, due to data filtered out for technical reasons. **(B)** Survival. The position of eight declared QTLs marked by gold circles. **(C)** Log drop boundaries on chromosome 11. QTL unique to growth or survival can be seen (e.g., Survival 401-419 kb; red arrow on B).

A chromosomal region with SNPs having FDR ≤0.2 was declared as a QTL with a 1 log-drop procedure defining boundaries as in Lipkin et al., 2016 (**Figure 3**). On this criterion, 51 growth and 96 survival QTLs were identified. Twenty-one of the QTLs overlapped between the two traits (**Supplementary Data Sheet 3**). **Supplementary Table 5** shows the distribution of the QTLs and the number of positive (increasing tolerance) and negative (decreasing tolerance) alleles for each parent, by chromosome location. QTLs were found on most chromosome; and for any single parent, positive and negative alleles were interspersed. The null hypothesis is that the only genes affecting ethanol tolerance are those that are responsible for the small observed difference in resistance between the more resistant and more susceptible parents. Clearly the two parental lines differ at many more loci than needed for this. Furthermore, on the null hypothesis, we would not observe transgressive variation in the F6. But such variation is clearly observed (**Supplementary Figure 5**). Thus, the two parental lines contain a very large amount of genetic variation that is not apparent when their mean values are compared, but which comes to expression in the cross (**Supplementary Figure 5**).

Mapping resolution based on the 1 log-drop boundaries was very high, yielding QTLs smaller than commonly found. Size of the QTLs averaged 11.2 kb (0.02–69.2 kb) for growth and 12.8 kb (0.001–54.8 kb) for survival (**Supplementary Data Sheet 3**). The distance between QTLs on the same chromosome averaged 350.0 kb (29.0–1,359.7 kb) and 186.2 kb (3.3–917.2 kb) for growth and survival, respectively. Thus, QTLs as close as 3.3 kb were distinguished. **Supplementary Table 6** shows the overall distribution of significant mapping-SNPs; indels among QTLs, ORFs, and regulatory sites; and distribution of significant SNPs according to effect on amino acid composition: synonymous and nonsynonymous substitutions, and regulatory sequences. The proportion of significant indels relative to significant SNPs among open reading frames (ORFs) (0.034) is much less than among QTLs overall (0.100). Of the SNPs in (ORFs), 64.5% resulted in synonymous substitutions, 35.5% in non-synonymous substitutions.

**Figure 4:**
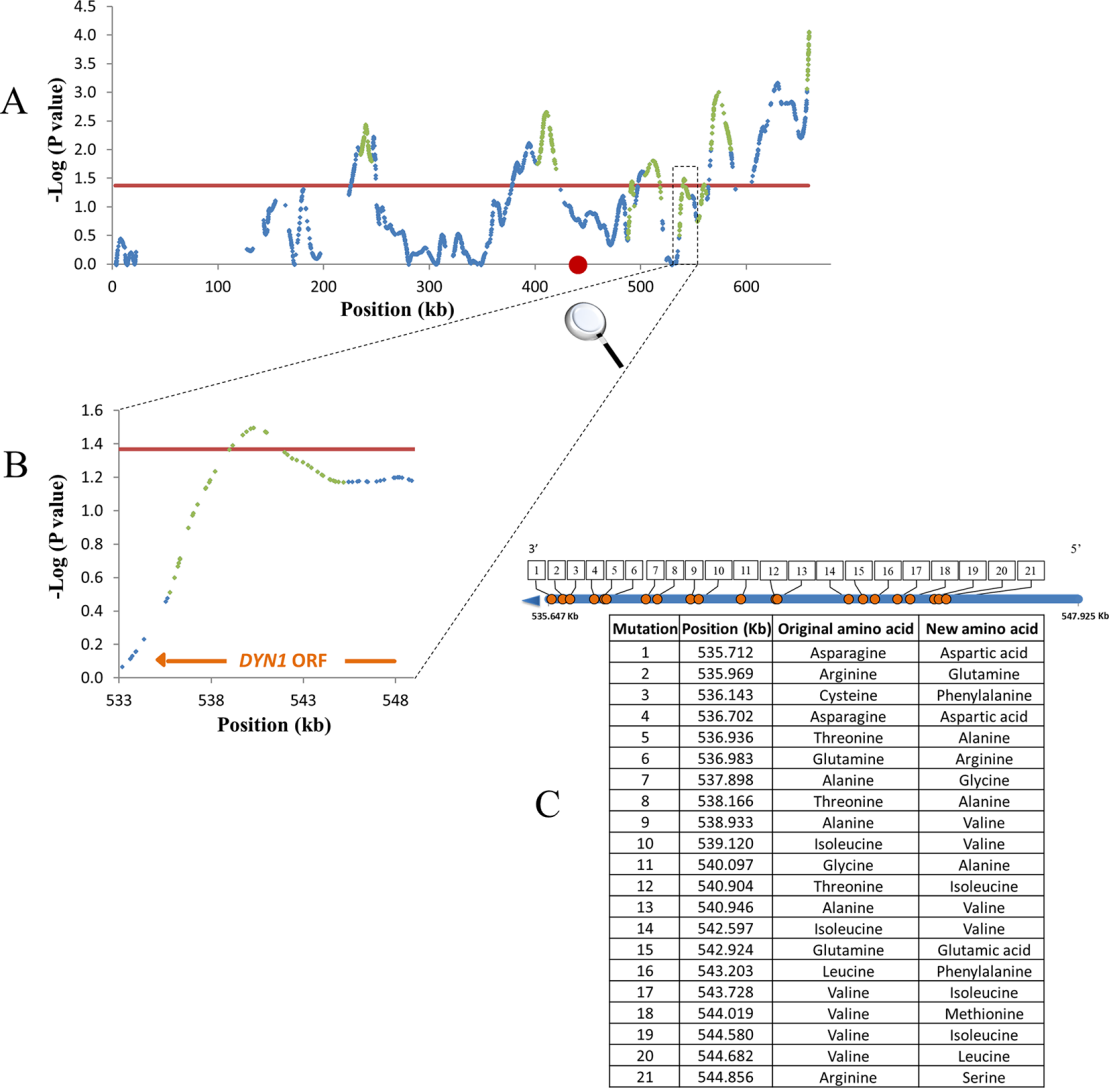
Example for mapping resolution to a single gene level. Shown are QTLs of survival trait on chromosome 11. Blue dots, smoothed SNP -log P values; red line, 0.2 FDR; Green dots, QTL log-drop boundaries; red circle, centromere. **(A)** Smoothed P-values. **(B)** Zoom-in on a small QTL containing part of *DYN1* gene. **(C)** Non-synonymous mutations in *DYN1*. Blue line, *DYN1* ORF; Orange circles, serial number by location of non-synonymous mutations. Mutation positions and alternative amino acids are summarized in the table.

Three of the survival QTLs and two of the growth QTLs did not include any ORF. These QTLs were quite small. For the two growth QTLs: QTL8 = 1,439 bp, QTL9 = 704 bp; for the three survival QTLs: QTL21 = 753, QTL30 = 967, QTL47 = 1,868. (**Supplementary Data Sheet 3**). All QTLs with no ORFs included SNPs located in various feature types (**Supplementary Data Sheet 4**). For both survival and growth, 10% of QTLs included only a single gene (10 and 5 QTLs respectively) (see an example in **Figure 4**), while 25% of survival and 33% of growth QTLs included more than six genes (**Supplementary Table 7**).

**Figure 5:**
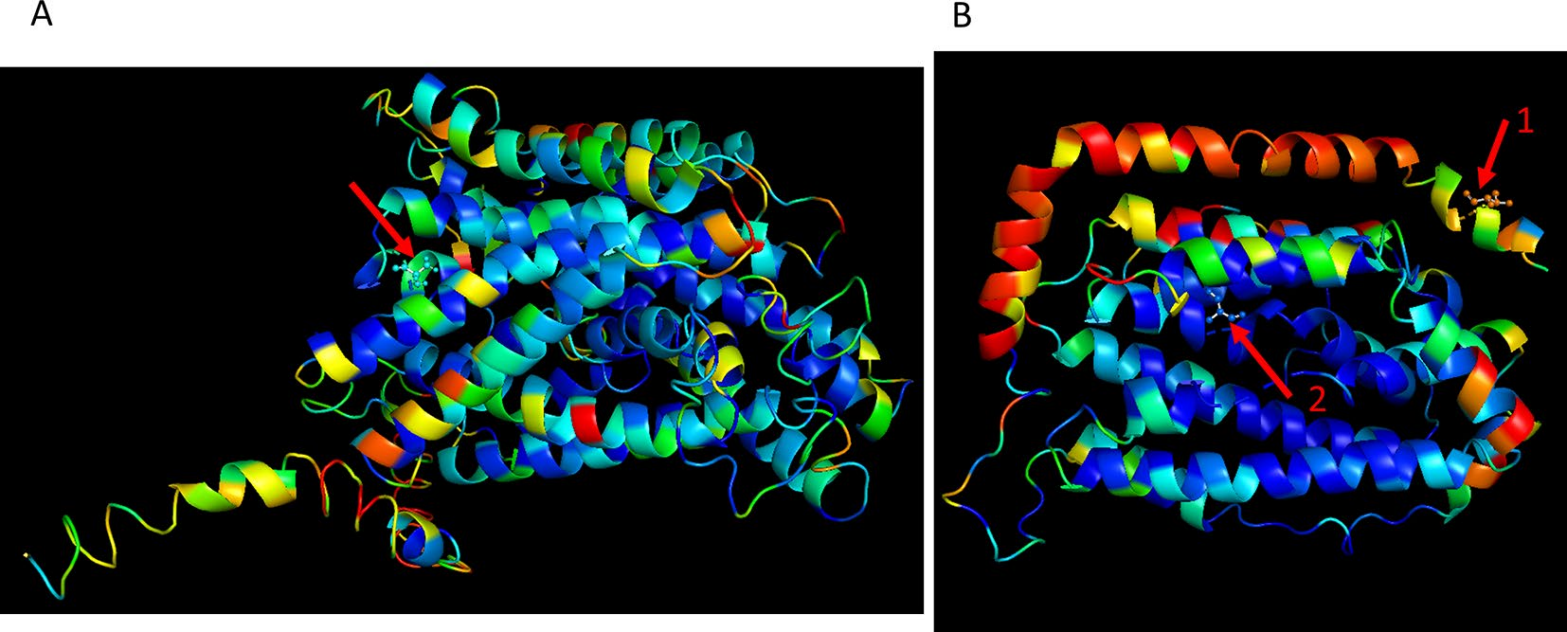
Examples of strong candidate mutations located in a QTL, presented in three-dimensional (3D) protein structures. Color scale represents amino acid conservation scores as defined by ConSurf software (Ashkenazy et al., 2010; Celniker et al., 2013): (Blue- conserved, Red- variable). **(A)** A candidate causative mutation in *MMP1* mapped to survival QTL 63. The mutation site at the red arrow (chr 12: 18,377; **Supplementary Data Sheet 4**), is located in an alpha helix conserved region. The substitution of GCC for GTC caused an alteration of Alanine to Valine. A destabilizing effect of this mutation on the protein, was predicted by DUET in five predicted models (Pires et al., 2014). **(B)** Two candidate causative mutations in *RNR2* (PDB: 1SMQ), mapped to growth QTL 22. The mutation site shown by red arrow 1 (chr10: 392,498; **Supplementary Data Sheet 4**) is located on the protein surface in a non-conserved region. The substitution of ACA for AAA caused an alteration of Threonine to Lysine. This mutation has high probability of affecting protein-protein interaction, as predicted by ODA (Fernandez-Recio et al., 2005). The mutation shown by red arrow 2 (chr10: 393,112; **Supplementary Data Sheet 4**) is located in a highly conserved region. The substitution of TCC for GCC caused an alteration of Serine to Alanine. A destabilizing effect of this mutation on the protein was predicted by DUET (Pires et al., 2014).

Since genotyping was by deep sequencing, the actual sequence of most polymorphisms is known. Therefore, we were able to propose candidate causative mutations. Lists of 3,943 and 7,936 mutations in QTLs for growth and survival, respectively, and alternative predicted proteins are given in **Supplementary Data Sheet 4**. Among the mutations found, some involved amino acid changes that are likely to alter protein activity. For example, *RNR2 *(in Growth QTL 22) and *MMP1 *(in survival QTL 64) were mapped to level of a single gene (**Figure 5**). For *RNR2* the known protein structure PDB: 1SMQ (Sommerhalter et al., 2004) was used. One mutation in* RNR2* (chr10: 392,498; **Supplementary Data Sheet 4**), located on the protein surface in a non-conserved region, caused an alteration of Threonine to Lysine. This mutation has high probability of affecting protein-protein interaction, as predicted by optimal docking area (ODA) (Fernandez-Recio et al., 2005). A second mutation in this protein (chr10: 393,112; **Supplementary Data Sheet 4**), located in a highly conserved region, caused an alteration of Serine to Alanine. A destabilizing effect of this mutation on the protein was predicted by DUET (Pires et al., 2014). For *MMP1* protein, as there was no known structure, we predicted it by several models. The percentage identity of the sequence in the structurally aligned region (Zhang, 2008; Roy et al., 2010; Yang et al., 2015) of the five most confidently predicted models was high and all gave similar predictions. A candidate causative mutation in *MMP1* (chr 12: 18,377; **Supplementary Data Sheet 4**), located in an alpha helix conserved region, caused an alteration of Alanine to Valine. A destabilizing effect of this mutation on the protein was predicted by DUET (Pires et al., 2014).

### Many QTLs of Small Effect Contribute to Phenotypic Variation

**Figure 6:**
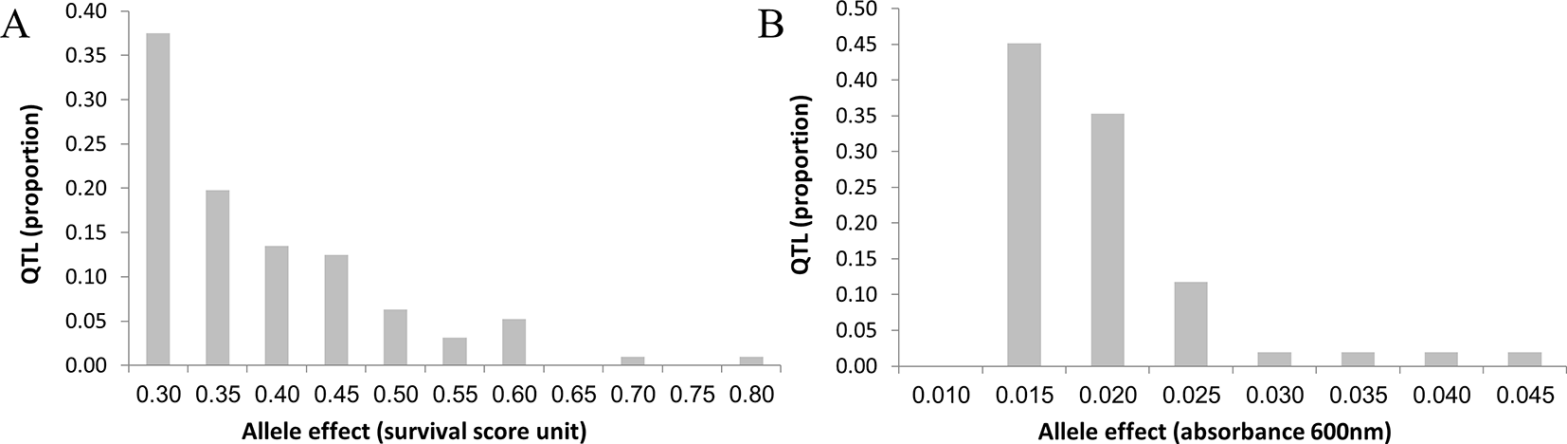
Distribution of QTL allele effects for survival and growth. The average effects for the three top markers in each QTL was used to estimate the QTL effect (the X axis). The distributions differ in detail, but both show complete absence of QTL of large effect, very low frequency of QTL of moderate effects, and a very large number of QTL of small effect. Thus, distributions differ from the infinitesimal model, in that the almost all variation is in QTL of small, but mappable effect; but also differ from typical QTL model in the complete absence of QTL of large effect, and paucity of QTL of moderate effect. **(A)** Survival. **(B)** Growth.

Allele effects averaged 0.018 (0.012–0.044) optical density (OD) units for growth and 0.357 (0.207–0.794) survival score units for survival (**Supplementary Table 8**). **Figure 6** shows the distribution of QTL-effects for growth and survival. For both traits, the distribution observed, namely, complete absence of QTLs of large effect (i.e., QTL individually explaining >5% of phenotypic variance); very small number of QTLs of moderate effect (i.e., QTL individually explaining 2% to 5% of phenotypic variance); and the remainder made up of QTLs of small effect (individually explaining <2% of phenotypic variance), is rather atypical for quantitative trait. Mean and range of contribution of individual QTL to the phenotypic variance were essentially the same for the two traits, namely, 0.7% (0.3–3.1%) for growth and 0.7% (0.3–3.3%) for survival. The total QTL-contribution to the phenotypic variance was, 34.7% and 72.0% for growth and survival, respectively. The total QTL-contribution to the *genetic* variance may be considerably greater. If, for example, heritability of the traits is 0.5, then the mapped QTLs would account for 69% (34.7/50.0) of genetic variation for growth and essentially all genetic variation for survival.

### Reciprocal Hemizygosity Analysis Strongly Supports the Mapping Results

For validation we investigated a few of the most likely candidate genes (mapped down to a single gene or due to their biological function) that were also located in genomic sites enabling simple gene manipulation. Based on mapping, we hypothesized that a candidate mutation generating a phenotypic difference between a tail and F6 control (i.e., on F6 genetic background) should also cause a difference in the same direction, between the two alleles in an F1 background on a reciprocal hemizygosity analysis (RHA) test (Steinmetz et al., 2002).

Four of the 10 gene × trait combinations differed significantly in the RHA (**Supplementary Figures 6**, **7**; **Supplementary Table 9**). Of the six remaining RHA, one (*NTH2* × survival) gave exactly equal results, while five differed in the expected direction, but did not reach statistical significance. This may simply be a matter of Type II error.

All nine differing RHA comparisons had the same direction as the QTL mapping (**Supplementary Table 9**). Thus, applying a sign test, the null hypothesis that the favorable alleles identified by QTL mapping and the favorable alleles identified by RHA are independent is falsified at P < 0.0002. Hence, at least some and most likely all of the RHA comparisons which did not reach significance can be taken to represent Type II errors, so that overall support of the QTL results by the RHA is very strong.

The effect of* ADH2* was validated by RHA for growth, while the positive allele was contributed from YE-531 (**Supplementary Table 9**). Since *ADH2* is an alcohol dehydrogenase which oxidizes ethanol to acetaldehyde (de Smidt et al., 2012), the identified positive allele is expected to reduce ethanol production. Indeed, we showed greater ethanol production of the YE-531-allele’s deletion strain in the F1 background, relative to its reciprocal deletion in the F1 (**Supplementary Figure 8**). By knocking out both copies of the *ADH2* allele, ethanol production was greater than in the F1 and reciprocal deletions. Importantly, initial glucose levels were equal for all samples (6.68%(w/v)). At the end (after 17 h of incubation), for all samples, glucose was not completely utilized so that glucose levels were not a limiting factor.

## Discussion

### QTL Mapping: Methodologies

The key to unlocking the full potential of the yeast model for genetic analysis of quantitative traits was the development in our laboratory (Bahalul et al., 2010) and elsewhere (Parts et al., 2011) of improved ascospore isolation procedures that increased the number of segregants that could be obtained in a yeast cross by orders of magnitude. Based on this, the present study was able to map to high resolution numerous QTL of relatively small individual effect. This was achieved by combining various advanced mapping designs, high-throughput sequencing methods, and statistical procedures—some well-known, others relatively recent into a “High Resolution Mapping Package” (HRMP). The HRMP includes AIL design to increase mapping resolution (Darvasi and Soller, 1995); two-stage individual phenotyping to increase trait heritability; genotyping by high-throughput sequencing so that most causative mutations are included in the analyzed markers; use of selective DNA pooling to enable low cost “genotyping by deep sequencing” of many individuals; LOESS smoothing of allele frequencies to reduce error variation (Chambers and Hastie, 1992); Log Drop analysis to define QTL boundaries (Lipkin et al., 2016); and setting significance thresholds by use of FDR rather than Bonferroni criteria. Foremost among these was the use of an AIL design, used here for the first time for mapping QTLs affecting ethanol tolerance. Multiple recombination events accumulated across the six AIL generations resulted in a fine-grained mosaic genome. This was demonstrated by genome sequencing of a single F6 segregant, revealing narrow haplotype blocks, with a median length of 16.1 kb (**Supplementary Figure 9**; **Supplementary Table 10**; **Supplementary text**). In a comparable study, Cubillos et al., 2013, using a four-parent AIL F12, found median haplotype block length of 23 kb across many individual genomes. The observed difference can be attributed to any number of biological or technical details that differ across the two experiments, e.g., difference in recombination rate of the founder strains and in the method used for AIL creation. Reflecting the narrow haplotype blocks, we obtained median QTL-length of 9.4 kb and 10.3 kb for growth and survival QTLs, respectively. This is directly comparable to 6.4 kb median QTLs length previously achieved by a 2-parent 12-generation AIL (Parts et al., 2011), as increasing the number of generations would decrease QTLs width proportionately.

Genotyping by deep sequencing ensured that causative mutations are included among the tested markers (except for sites with low sequencing reliability such as repetitive elements), minimizing occasional loss of power of closely linked markers due to hotspots of recombination or sampling variation in proportion of recombination between marker and causative mutation (Soller et al., 1976). Sequencing pools allowed deep sequencing at exceptionally high coverage (about 1,000-fold), of larger samples than feasible by individual genotyping, thus increasing statistical power. The two-stage combination of mass selection followed by replicated phenotyping of individual segregants prior to pooling made optimum use of the ability of yeast to increase selection intensity by testing a very large number of segregants and improved selection accuracy by replicated phenotyping of vegetatively produced daughter colonies (Soller and Beckmann, 1990). In this way, both the genetic difference between the tails and the statistical power of the experiment were increased. The 90 segregants that were genotyped for each trait represent selected 10% of the starting population; thus they represent mapping power of a much larger number of segregants (Darvasi and Soller, 1992).

Local smoothing by LOESS (Chambers and Hastie, 1992) eliminates outlier marker frequencies that do not fit the frequency of their close environment and reduces sample noise (**Supplementary Figure 10**). In this way, LOESS reduced error variation of frequency and variance estimates of each SNP. Smoothing also enabled use of the convenient log-drop method for setting boundaries of QTLs (Lipkin et al., 2016).

Finally, the use of FDR criterion to set critical thresholds for significance greatly reduced Type II error while controlling Type I error, in this way enabling many more true effects to be uncovered compared to use of a Bonferroni criterion (Benjamini and Yekutieli, 2005).

### Numerous Mapped QTLs of Small Effect Made a Substantial Contribution to the Phenotypic Variation

Using the above package of methodologies to map QTLs affecting yeast performance under ethanol stress, we found 51 QTLs affecting growth and 96 QTLs affecting survival. Of these, 21 affected both traits. The extent to which tolerance QTL are specific to a single trait, or are general to a number of traits, is of course of great practical and scientific interest. We expect to have at least some overlapped QTLs since both of the traits are aspects of ethanol tolerance and of general resilience in the face of stress. In addition, overlapping QTLs may be due to the initial survival-selection of the segregants. Finally, it should be noted that statistical power of the experiment, although high, was not complete. Consequently, some QTLs that appear to affect only one of the traits, e.g., survival, may actually affect both, but due to sampling variation, only the effect on one of the traits was statistically significant and was not counted as an overlapping QTL. Disentangling all of these possibilities is not within the scope of this paper. Thus, there were a total of 126 QTLs affecting the two tolerance traits. This is by far the largest number of QTLs identified in any single QTL mapping study in yeast for any trait. Compare, for example to the studies by Ehrenreich et al., 2010 and Bloom et al., 2013, using SDP in a large mapping population generated by a cross between a laboratory strain and a wine strain. The former mapped 17 chemical resistance traits; maximum number of QTL for a single trait was 27, median 12. The latter mapped 46 quantitative traits in the same population; maximum number of QTL for a single trait was 29, median 12. In addition, our results are more than an order of magnitude greater than found in previous QTL mapping studies for ethanol tolerance: Swinnen et al., 2012a, identified three QTLs; Ehrenreich et al., 2010, 2; Hu et al., 2007, 5; and Pais et al., 2013, 19, of which 11 QTLs were identified in one population, and eight QTLs in a second population. We attribute the much larger number identified in the present study to a number of factors: (1) The nature of the cross which apparently captured a large part of the natural genetic variation in the trait, (2) high statistical power that allowed mapping the many dozens of QTLs of small effect segregating in the population, and (3) the use of an FDR criterion to set critical thresholds for significance, which reduced the very high Type II errors that obtain when a Bonferroni criterion is used. Given the complexity of the trait, these large numbers of QTLs are biologically plausible, and accord with estimates of hundreds of QTLs affecting height in humans (Visscher, 2008; Manolio et al., 2009; Visscher et al., 2012; Wood et al., 2014), and dozens of genes affecting bristle number in *Drosophila* (Mackay and Lyman, 2005) and flowering time in maize (Buckler et al., 2009). Buckler et al., 2009 noted that they uncovered numerous QTL of small effect but not even a single QTL of large effect. This is similar to our mapping results (**Figure 6**).

QTL effects averaged 0.018 OD units for growth and 0.357 survival-score units for survival. It is convenient to express these effects in units of the phenotypic standard deviation (standard deviation units, s.d.u.), so that they can be compared across traits (SD = 0.148 OD units for growth; 2.75 survival units for survival). Expressed in this way, QTL effects for the two traits were virtually identical: average effect = 0.121 s.d.u. for growth (range 0.081 to 0.295), and 0.129 s.d.u. for survival (range 0.075–0.288). These values are small, but not negligible. To put them in perspective, average QTL effect was 2.6% of the mean for growth) (0.698 OD units), and 4.5% of the mean for survival (survival-score units). Thus, the cumulative effect of 50 QTLs for growth or 25 QTLs for survival would double the mean growth and survival scores, respectively. On average, the identified QTLs individually explain about 0.7% each, of phenotypic variation. Taken together, they account for a substantial fraction of the phenotypic variation, 34.7% and 72.0% for growth and survival, respectively. The proportion of genetic variation explained would be even greater, depending on the heritability of the trait. Taking heritability = 0.50, for example, the mapped QTL explains almost 70% of genetic variation in growth and essentially all of the genetic variation in survival.

### The Distribution of QTL Effects for Both Traits Represent the “Quasi-Infinitesimal” Genetic Architecture of Ethanol Tolerance

A number of very large-scale QTL mapping experiments explored the architecture of complex quantitative traits, using yeast as a model system. Each study involved numerous traits, mainly ability of yeast to grow under various adverse conditions (e.g., high temperature; toxins) (Cubillos et al., 2011; Bloom et al., 2013; Hallin et al., 2016). Generally, less than 20 QTLs were detected for any given trait, while all QTLs taken together explained 10–80% of the trait phenotypic variance. The results of the present study differed from the model studies in the much greater number of QTLs uncovered, and in the absence of QTLs of large effect (i.e., QTLs accounting individually for more than 5% of phenotypic variation) and very small number of QTLs of moderate effects (i.e., accounting individually for 2–5% of phenotypic variation). Thus, almost all genetic variation is due to the very large number of QTLs of small effect. We can term this a “quasi-infinitesimal” model, in which genetic architecture consists of a large number of loci of small effect, but the effects are detectable effect under appropriate circumstances and are individually due to single causative mutations. We can contrast this to a full infinitesimal model where the mapped effects are due to cumulated effect of multiple causative mutations of almost infinitesimal effect (Soller et al., 1979; Visscher and Haley, 1996). Whether the observed results of our study represent a quasi or full infinitesimal model cannot be resolved by the data presented here alone. On average, the QTL regions for growth and survival included median 65–68 polymorphic sites per QTL, of which 17% (about 10) are predicted to change protein sequence and therefore have the potential to affect phenotype. However, we believe that the large number of QTL in yeast that has identified the causative mutation (Winzeler et al., 1998; Deutschbauer and Davis, 2005; Gerke et al., 2009), including the results of the present study, mitigates against a true infinitesimal model.

Generally, the model studies uncovered a much higher proportion of loci of moderate to large effect compared to the present study. This may represent a real difference in the genetic architecture of ethanol tolerance in the wild, which is under strong natural fitness-selection for an optimum that would tend to eliminate alleles of large effect, and the genetic architecture of the model traits studied by Cubillos et al., 2011, Bloom et al., 2013, and Hallin et al., 2016, which for the most part are laboratory artificial traits and not related to natural evolutionary fitness.

Interestingly, previous mapping studies by Hu et al., 2007 and Cubillos et al., 2011, each based on F2 of a cross between two yeast strains that differed greatly in ethanol tolerance, uncovered five and one QTLs affecting ethanol tolerance, respectively. In Hu et al., 2007, one QTL was of large effect accounting for 25% of phenotypic variance in the F2. and four, of moderate effect accounting for 3% to 9% of F2 phenotypic variance. In that study, the methodology for scoring survival was similar to the methodology in the present study. In Cubillos et al., 2011, the QTL were mapped by measuring growth under 7% ethanol. A single QTL of large effect, accounting for 36.5% of phenotypic variance in the F2, was identified. Thus, in both studies the overall distribution of effects was the complete opposite to that found in the present study. This can be attributed to the different biology and ecological genetics of the strains involved. In the present study, two parental stains of moderate tolerance, the standard laboratory strain and a strain isolated from nature, that differ only slightly in ethanol tolerance were used (**Supplementary Figure 2**). In the cited studies, two widely differing strains at opposite ends of the tolerance spectrum were used. Within this context, it is encouraging to note that a number of candidate genes within the mapped QTLs of Hu et al., 2007, were found in the ethanol tolerance QTLs of this study: *MSK1 and APJ1* were found in growth QTLs; *PIC2, GIP2,* and* SSA4* were found in survival QTLs; *PFK26* was found in QTLs of both traits (**Supplementary Data Sheet 4**). We also identified the DNA binding site for the product of *SWI4* gene, which was a candidate in Hu et al., 2007. *APJ1* was also found among six genes previously mapped and validated by RHA (Swinnen et al., 2012a; Pais et al., 2013).

Several of the genes mapped in our study share biological pathways that were linked to ethanol tolerance trait by an experimental evolution study (Voordeckers et al., 2015). Of these, *SIC1, CDC53*, and *HOS3* are involved in cell interphase; *MDS3* and *PKC1* participate in pathways related to cell cycle; and *CSE1* and *VPS70* have a role in protein transport. These findings strengthen the reliability of the mapping results. In addition, many of the candidate mapped genes identified in the present study were previously linked to ethanol tolerance by biological interaction analyses, gene knockout studies, and other non-mapping studies.

### Biological Context of Identified QTLs

The high resolution in the present study helps to directly identify candidate causative genes; as among the many mapped QTLs, 10% contained only one gene. **Figure 4** shows an example for mapping resolution to a single gene level, for the *DYN1* gene that functions in membrane trafficking, and spindle position required for cell segregation in yeast (Eshel et al., 1993).

In addition, some of the QTLs include mutations that are likely to have a direct phenotypic effect. Such mutations are those that involve early stop codons, mutations in conserved regions, frameshift mutations, and more (**Supplementary Data Sheet 4**). Indeed, in the growth and survival QTLs, 2 and 10 mutations, respectively, involved substitution of an amino acid by an early stop codon; while in 4 and 11 mutations, respectively, a stop codon was changed to an amino acid (in respect to the sequence of S288c). Several mapped mutations were predicted to have a strong effect on protein structure and function of two genes (*MMP1* and *RNR2*) mapped to a level of a single gene (**Figure 5**). For *MMP1*, an S-methylmethionine permease (membrane transport protein) (Rouillon et al., 1999), a non-silent mutation substitution located in an alpha helix-conserved region, was predicted to reduce the stability of the protein. Since membrane function and structure interference under ethanol stress is well known (Sikkema et al., 1995), the predicted effect of *MMP1* on ethanol tolerance is not surprising. *RNR2*, the second investigated gene is a ribonucleotide reductase which is regulated by DNA replication and DNA damage checkpoint pathways (Elledge and Davis, 1987). Here, two non-silent mutations were mapped: one located on the surface of the protein in a site predicted to affect protein-protein interactions and the other located in a highly conserved region and predicted to reduce the stability of the protein.

The mapped genes: *ADH2, MOG1, MGS1,* and YJR154W, were validated by RHA. *ADH2* is an alcohol dehydrogenase whose primary function is to oxidize ethanol to acetaldehyde (de Smidt et al., 2012). *MOG1* is involved in nuclear transport mechanism (Oki and Nishimoto, 1998), known to protect the cell from stress induced damage (Saavedra et al., 1996; Takemura et al., 2004). *MGS1 *is related to DNA replication stress (Tkach et al., 2012). YJR154W is uncharacterized.

For *ADH2*, the positive allele was contributed from YE-531 (**Supplementary Table 9**). However, since *ADH2* is an alcohol dehydrogenase, the same allele is expected to reduce ethanol production. We validated that expectation, showing increased production of ethanol by the YE-531-allele deletion strain in the F1 background, relative to its reciprocal deletion and the F1 (**Supplementary Figure 8**). Also, by knockout of both copies of *ADH2* alleles in the F1 background gave highest ethanol production relative to the other tested lines, as also shown by previous studies (Ida et al., 2012; Ye et al., 2016).

### QTL Mapping and Genetic Improvement of Ethanol Tolerance

We uncovered over 100 QTLs affecting ethanol tolerance. As the QTL mapping association tests were single marker tests in a segregating population, the QTL effects as measured in this population must be additive, at least in part, i.e., do not depend for their expression on genetic interactions or genetic background. This means that the overall mean phenotypic value of a line is given by the sum of the positive and negative effects across all QTL. Consequently, the phenotypic value of the line will depend on the balance of positive and negative alleles at the QTLs affecting the trait value. Thus, two lines can have very similar mean phenotypic values yet differ greatly in detailed genetic composition. This is nicely illustrated in the results of the present study. The distribution of positive alleles for each parent across the genome was almost equal but differed in the specific set of QTLs providing the positive and negative alleles. Thus, our study revealed that although the two parental strains have similar mean phenotypic values (**Supplementary Figure 2**), they contain an enormous amount of genetic variation that is hidden from view when parental phenotypes are compared.

Crossing two parent lines of this nature to create a so-called “synthetic” population, with independent assortment and recombination, releases this genetic variation for selection (**Supplementary Figure 5**). Segregants of the following generations that have a higher proportion of positive (or negative) alleles than either of the parent lines, manifest as “transgressive variation”, exceeding both high (or low) parent lines in trait value. Of greater importance, recurrent selection for high trait value in the synthetic population will lead to ever higher frequency of positive alleles, generating genotypes and performance levels that were never previously present in the species. Indeed, modern highly selected farm animals achieve genetic performance levels that were never found in the original parental populations prior to selection. It is not far-fetched to expect that the same will hold true for ethanol tolerance or other performance traits in industrial yeasts, conditional on avoidance of inbreeding (Hill, 2016).

### QTL Mapping for Marker or Gene Assisted Selection

Selection based on phenotypes as described above requires massive investment in evaluating phenotypes. QTL mapping can substitute for at least part of this effort, by providing markers that quickly, cheaply, and reliably identify the individuals carrying the most positive alleles for the target trait, a process termed “Marker Assisted” or “Gene Assisted” selection (MAS/GAS). MAS/GAS is particularly important when phenotyping costs are high or heritability of the trait is low, so that simple phenotypic selection is inefficient.

Genetic improvement can be attained by introgressing the positive alleles of the mapped QTLs from one parent to the other. In the past, this could only be done by laborious and time consuming multiple marker crossing and selection introgression designs that are limited to a small number of genes at any one time (Koudande et al., 2000). In budding yeast, direct introduction of positive alleles from one parent line to another, can be achieved by genome engineering, using homologous recombination or CRISP/Cas9 methodologies. It has been suggested that any single genetic modification is not likely to improve yeast strains for ethanol tolerance and high production, since these are complex polygenic quantitative traits affected by numerous loci of small effect (Stanley et al., 2010; Swinnen et al., 2012a; Lam et al., 2014). While this is undoubtedly true for a single locus, the stacked effects of numerous loci of small effect can add up to a large total effect on line performance.

Finally, identifying causative QTG and Quantitative Trait Nucleotides (QTN) underlying genetic variation at a QTL, may also identify the causative pathway in which the QTG is operative. This may open further opportunities for improvement by reverse engineering of other genes in that pathway.

## Materials and Methods

### Intercross

The haploid laboratory strain S288c, and a haploid strain YE-531 isolated from nature (Ezov et al., 2006), were crossed to create an F1. The cross population was intercrossed for five more generations to create the F6 AIL population, using the Ether-zymolyase (EZ) ascospore isolation procedure (Bahalul et al., 2010). This procedure combines two conventional protocols, enabling a major increase in the efficiency of ascospore isolation. Live count by plating was carried out to determine the effective number of segregants, which was >10^5^ each generation. This amount greatly exceeds the 100 individuals per AIL generation, recommended to minimize sampling effects on recombination proportions across the genome (Darvasi and Soller, 1995).

### Pool Construction

Three independent aliquots from the unselected AIL F6 population (more than 3.5 × 10^5^ segregants/ml) served as the control group. We studied survival under high ethanol stress and growth under moderate ethanol stress as two distinct traits. To establish the tolerant tail pools for the two traits, a two-stage selection was employed in the F6 generation (**Figure 2**). The first stage consisted of mass-selection at an ethanol concentration that provided 35% survival (based on CFU of the unselected and challenged populations). This level of survival was chosen as one that would provide significant selection for ethanol tolerance while maintaining genetic variation in the selected group. The 35% survival target was obtained by exposure to 15% (V/V) ethanol stress for 5 h. At the end, 300 segregants were randomly sampled among the thousands of survivors.

In the second stage, the selected 300 segregants were individually evaluated for growth and for survival under ethanol stress (**Supplementary Data Sheet 5**). Anaerobic conditions were obtained using a polyester-based microplate film, designed to minimize evaporation (USA Scientific). Each segregant was evaluated in four replicates for each trait as follows. Two aliquots were sampled of each segregant culture. An equal number of cells, relying on OD measurement, was taken from each aliquot, to which we added media with ethanol. Then we divided each media-ethanol aliquot into two, giving a total of four replicates.

For identification of the segregants to make up the selected growth upper tail, 1 ml final volume of 0.2 OD cell culture suspended in YPD liquid media mixed with ethanol, was transferred from each replicate into one well of a 24 multiwell plate. The ability of the replicate to grow under stress of 9.5% (V/V) ethanol for 22 h was tested by final OD600 nm measurement. Average and standard deviation of final OD600 nm values of the four replicates were calculated for each tested segregant (**Supplementary Figure 5A**).

To test for ethanol degrading ability, the ethanol concentration of these cultures was determined by Headspace GC-FID (Gas Chromatography with Flame Ionization Detector), before and after overnight growth for 22 h. Ten segregants suspected of having ethanol degrading activity were excluded. Of the remaining 290 segregants, the top 90 showing the highest OD values were chosen to form the upper tail.

For the creation of the survival upper tail group, the ability to survive in 19% (V/V) ethanol solution for 5 h was tested by a spot assay. Namely, for each replicate, 1 ml final volume of 1 OD cell culture suspended in YPD liquid media mixed with ethanol, was transferred into one well of a 24 multiwell plate. A spot assay at 1- to 100,000-fold dilution was used (in YPD plates, with no ethanol) to evaluate cell survival ability. The survival-ability test was designed to test the ability to survive for a short time (5 h) under high ethanol stress. Survival score was assigned depending on dilutions showing survival in the spot assay, and selection was based on total score across all four repeats (0–14; 0–no survival ability, 14–high survival ability) (**Supplementary Figure 5B**).

The 300 segregants selected in the first stage were ranked on basis of OD in the growth test and survival score in the survival test, and the top 90 (30%) most tolerant segregants for each trait were chosen for the upper tail. For the statistical analysis (below), for each of the two traits, the 90 selected segregants were divided at random into three independent groups of 30 segregants each.

Importantly, although only 90 segregants were eventually taken for genotyping and mapping; selection proportions in the tails were 35% and 30% in the first and the second stage; together 10.5%. However, as the design used here was a 1-tail design (unlike Darvasi and Soller, 1994, 2-tails design), average selection was equivalent to 21%. Thus, on principles of SDP, these 90 tail segregants are equivalent to a mapping population of about 429 unselected F6 segregants, with respect to mapping power for a trait where both tails are included in the analysis.

### Preparation of DNA Samples

Genomic DNA from founder strains and pools was extracted with MasterPure yeast DNA purification kit (Epicenter, Madison, WI), according to the manufacturer’s instructions (see more details in **Supplementary text**).

### Genotyping by Whole-Genome Deep Sequence Pool Analysis

Sequencing by Illumina HiSeq 2500 technology was performed with paired ends of 100 bp fragments for parental strains and pools. Genome coverage (the number of unique mapped reads multiplied by the read length, divided by the genome size), was around 1,000 for all pools (**Supplementary Table 11**). DNA reads were aligned to S288c reference genome sequence, version R64-1-1 downloaded from the Saccharomyces Genome Database (Cherry et al., 2012), and to a YE-531 reference genome that we generated by: 1) Initial assembly of YE-531 High Throughput Sequencing data using Edena assembler (Hernandez et al., 2008); and 2) Extending the contigs using AlignGraph (Bao et al., 2014) and the S288c genome. Sequence quality score >20 per base was confirmed using FastQC (Koudande et al., 2000).

Sequence alignment was done with BWA-mem algorithm, version 0.7.10 (Li and Durbin, 2009). Only unique mappings were used in the analysis. PCR duplicates were marked by Picard-Tools 1.123. Alignment manipulations, variant calling and allele frequency estimations were done by GATK best practice v.3.3-0 (McKenna et al., 2010). Suggested protocol with the default parameters was used. In addition to variant calling with GATK, we used MUMmer 3 package (Kurtz et al., 2004) variant calling to compare between S288c and YE-531 assemblies.

To confirm the allele frequencies obtained by deep sequencing, we individually genotyped two of the SNPs in individuals from the control and the tolerant groups, using High Resolution Melting (HRM) analysis (**Supplementary Figure 11**; **Supplementary text**).

### Quality Control

For QTL definition we used only SNPs agreed between GATK and MUMmer among all of the SNPs that were found through sequencing. Allele frequency of each variant was estimated in F6 population pool samples twice, based on S288c and YE-531 reference genomes separately. Variants that had different frequency estimates as obtained by the two reference genomes were removed from the analysis.

Since YE-531 is haploid, we expect that any allele that differs from S288c will have YE-531 frequency of 1 in pure YE-531 samples. Therefore, we removed markers with YE-531 frequency <1 in pure YE-531 samples, as they might reflect technical errors. In addition, markers having MAF <0.05 in pool samples were also removed from the analysis. After filtering, 35,134 SNPs for growth and 35,019 for survival (out of about 80, 000 SNPs) were used for mapping QTLs.

### Frequency and Variance Estimates

Allele frequency estimates were smoothed along each chromosome by location, using LOESS local regression with span (i.e. window) = 80 SNPs and degree = 2. We used the LOESS implementation in R that is based on cloess (Chambers and Hastie, 1992). The smoothed allele frequencies were used to calculate D-values (the frequency difference between the tail groups and the AIL F6 control groups). Then, SD among replicates was smoothed by allele frequency with span (bin size) = 10% of the SNPs and degree = 2 as adapted from Lipkin et al., 2016. Finally, variance among replicates was calculated as the square of the smoothed-SD.

### Statistical Analysis

#### Notation

Basically, we used a simple single-marker test for marker-trait association. That is, each marker, denoted Mi, was tested individually for association with growth, and separately for association with survival. Simply put, if frequency in the growth or survival pools of alternative alleles at a given marker differ more widely from their frequency in the control pools than expected by chance, we attribute this to association of the alleles at the marker with the tested trait. Thus, taking growth for example as the target trait, the following steps were taken for testing Mi for association with growth.

1. Estimate frequency of the alternative alleles of the target marker Mi in the growth pools (denoted, piG) and in the control pools (piC). (Since frequency of the S288c allele in a pool = 1-frequency of the YE-531 allele in that pool, either allele can be used as the basis for statistical tests. In the present study, all further calculations were based arbitrarily on the frequency of the YE-531 allele of the marker).
2. Calculate the observed difference, denoted Di = piG − piC, in marker allele frequency between the pools of the target trait (in our case the growth pools) and the control pools.
3. Obtain the expected difference (termed the “standard error” of Di), denoted SE(Di) under the null hypothesis H_0_: marker is not associated with trait value (see later for how SE(Di) was obtained).
4. Calculate Zi = Di/SE(Di). If Di is much greater than SE(Di), such that Zi >1.645, we conclude that Di was not generated under the null hypothesis. i.e., the null hypothesis is falsified, and the alternative H_1_: there is an association between marker and trait, is accepted; with possibility of error (i.e., falsifying H0 when H0 is true) = 0.05. This is termed a CWER, as it is error rate for a single comparison. If one tested many markers all located on a single chromosome, then we could have “chromosome-wise error rate”, which would be the error rate of having even one of the markers on the chromosome falsely associated with trait value. Similarly, there could be an “experiment-wise error rate”.

The critical value of Zi = 1.645 for Zi to represent a CWER of P < 0.05 is obtained from tables of the cumulative standard normal curve.

(v) If Zi <1.645, we conclude that null hypothesis has not been falsified, and hence we are not justified in claiming that there is an association between marker and trait.

In applying these principles to our case, we need to take into account that the estimates of allele frequency in the selected pools and in the control are based on three subpools for each pool or control. The estimates of selected frequency in the selected pools and in the controls are thus based on average of the three subpools. Also, in our case, we do not use a standard error based on theoretical derivation, but an empirical standard error based on the variance among the frequency in the three subpools of the selected pools, and the three subpools of the control pool.

However, detailed examination of the any GWAS based on such single marker tests, showed that even significant regions are always a mixture of significant markers and non-significant markers, making it difficult to set boundaries for the QTL region (QTLR). To circumvent this, following Lipkin et al. (2016), we smoothed the results, in this study by the LOESS method, as detailed below.

The following is a more explicit presentation of the application of the above principles to our specific case.

Let pMijk be the LOESS smoothed frequency estimate (eFrequency) of the YE-531 allele of the i_th_ marker in the j_th_ pool of the k_th_ category,

where,

i = 1 to M markers used in the study,

j = 1 to 3, is the serial number of the random subpool within its category,

k = 1 to 3 is the category, where 1 = Growth selected pools; 2 = Survival selected pools; 3 = Control (F6) pools.

Thus, pM111 would be the eFrequency of Marker 1, in subpool 1, of the growth selected pools.

Marker-trait association was determined by a single-marker test, separately for growth and survival, where CWER P-value of the ith marker was set equal to

$$\begin{array}{l} \text{Pi = 2}\times \text{ area of the standard normal}\\ \text{ curve to the right of Zi = Di/SE(D)} \end{array}$$

where,

Di = Avg(pMi.1) − Avg(pMi.3), for the growth trait pools, and

Di.2 = Avg(pMi.2) − Avg(pMi.3), for the survival trait pools.

Avg(pMi.1) is the average of pMi taken across the three growth subpools. The dot in the expression Avg(pMi.1) tells us that the average is taken over the three growth subpools.

Avg(pMi.2) is the average of pMi taken across the three survival subpools,

Avg(pMi.3) is the average of pMi taken across the three AIL-F6 control subpools.

SE(D.k) is the standard error taken over all pools and markers in a given category.

By definition, the SE of a “treatment” effect is the expected standard deviation under the null hypothesis that the treatment (the above Di.k), has no effect. An empirical estimate of the SE(D.k) was obtained based on the assumption that the variance of marker allele frequency among pools of the same category (i.e., among growth, survival or control pools), is affected by sampling variation and technical variation, but does not represent a real difference in marker allele frequency due to a QTL effect. Thus, it is a measure of sampling variation among pools within a given category under the null hypothesis (Mosig et al., 2001; Korol et al., 2007; Bagnato et al., 2008). SE(D.k) was obtained by appropriate weighting of these variance estimates.

To implement this, for each marker we calculated the variance of the marker allele frequency across the three F6 control subpools. The locally smoothed variance was calculated to obtain VarPijk for each marker, an empirical estimate of the sampling variation among control subpools under the null hypothesis. Similarly, for each marker × trait combination, the variance of marker allele frequency across all three tail subpools was calculated. Then the locally smoothed variance across span of 10% of the markers for growth and survival pools, respectively (VarSijk.1 and VarSijk.2), was calculated.

Since Avg(pMi) for the various categories is an average of three subpools, SE2(Dijk) = VarSijk/3 + VarPijk/3, where Dijk = D for the ith marker, in the jth pool, of kth category.

### Declaring Significance and QTL Mapping

To account for the multiple tests involved in the present study (> 35,000 marker tests for each trait), we used the False Discovery Rate (FDR) criterion (Benjamini and Hochberg, 1995), with alpha = 0.2 to set experiment wide significance for the individual marker × trait combinations (**Figure 3**). The critical CWER P-values for declaring significance at FDR = 0.20, were 0.046 for growth and 0.033 for survival. This is quite a bit higher and much more permissive than the Bonferroni threshold. But, of course, this is exactly the reason we employed the FDR criterion. In addition, the Bonferroni threshold would require taking into account the correlation among closely linked markers while the FDR criterion holds under positive dependency (Benjamini and Yekutieli, 2001). Benjamini and Yekutieli, 2005 provided detailed and exhaustive justification for the use of the FDR criterion in QTL mapping.

QTLs boundaries were defined with a 1.0 log-drop support interval procedure as in Lipkin et al., 2016 (see also Lander and Botstein, 1989). Briefly, 1 log-drop procedure starts from the top log_10_P value in a region, and moving out on both sides until a drop of 1.0 of the log_10_P value is obtained (that is, if the top log value was 4.0, moving out until the first log_10_P ≤ 3.0). Note that a single QTL may be considered as two QTLs when two adjacent peaks are observed. We considered QTLs of the two traits as overlap when their 1.0 log-drop boundaries partly overlapped.

### Phylogenetic Analysis

We compared our strain YE-531, isolated from nature, and the popular laboratory strain S288c with a selected set of 13 commercial and wild strains available in the SGD database (Cherry et al., 2012). Some of the strains represent main lineages that appear in Liti et al., 2009; Schacherer et al., 2009; Maclean et al., 2017. Phylogenetic analysis was performed on the whole genome sequence of the 15 strains using RealPhy (Bertels et al., 2014) with default parameters except for the tree building. The tree building was performed with PhyML (Guindon and Gascuel, 2003) using non-parametric bootstrap 100 times. The tree was drawn with Dendroscope (Huson and Scornavacca, 2012), bootstrap values are presented on the branches in black. A radial phylogenetic tree is presented in **Figure 1**.

### Marker Allele Substitution Effect

Allele substitution effect (δ) was calculated for each marker located in a QTL following Darvasi and Soller, 1994, except that instead of twice the allele substitution effect (2δ), obtained by comparing two extreme tails, here δ was obtained by comparing each tail to the unselected F6 population (see a detailed example in the supplements). The phenotypic means of each tail and control F6 isolates were obtained from ethanol exposure experiments, similarly to those described in the second stage of the Pool construction. As detailed above, a two-stage selection scheme was used (**Figure 2**). Selection proportions in the tails were 35% and 30% in the first and the second stage, together 10.5%. However, as the design used here was a 1-tail design (unlike Darvasi and Soller, 1994, 2-tails design), average selection was equivalent to 21%.

### QTLs Allele Effect and Contribution to the Phenotypic Variance

The average of effects of the top three markers in each QTL was used to estimate the QTL allele effect, δ. The contribution of the QTL to the phenotypic variation was calculated as VarQ = 2pqδ^2^ (Mosig et al., 2001), where p and q are the means of allele frequencies of the same three markers used to estimate the QTL effect. VarP, the phenotypic variance of each trait in the entire F6 population, was obtained from ethanol exposure experiments, performed similarly to the second stage selection procedures, and the proportional contribution of the QTL to VarP was calculated as VarQ/VarP.

### Reciprocal Hemizygosity Analysis

For RHA (Steinmetz et al., 2002), deletions were made in the S288c and YE-531 haploid backgrounds (see more details in **Supplementary text**). Reciprocal strains were generated by crossing the deletion parental with the other parental strain. To determine whether one allele is advantageous over the other in the RHA tests, we tested growth and survival of the two reciprocal deletion strains under ethanol stress, as in the second stage of the pool construction. We tested six genes for a single trait only (growth or survival), and two genes for both traits, making a total of 10 gene × trait tests. More details regarding the tests and the RHA statistical analysis can be found in the **Supplementary text**, and in **Supplementary Table 9**.

### Testing Candidate Causative Mutations in Three-Dimensional (3D) Protein Structures

For gene with a known protein structure, the PDB data (Bernstein et al., 1977) were used. For proteins with unknown structure, we predicted the structure using the servers Phyre2 (Kelley et al., 2015) and I-TASSER (Zhang, 2008; Roy et al., 2010; Yang et al., 2015). The location of non-synonymous mutations on the 3D structure of tested proteins was identified. In addition, the level of evolutionary conservation based on each mutation was estimated using ConSurf software (Ashkenazy et al., 2010; Celniker et al., 2013); favorable sites for protein-protein interaction were detected by ODA (Fernandez-Recio et al., 2005); effects of mutations on protein stability was predicted by DUET (Pires et al., 2014).

## Author’s Note

This paper is dedicated to the memory of our colleague and dear friend Prof. Ariel Darvasi (1962–2018) who contributed so much to the development of the selective DNA pooling and AIL mapping procedures on which this study is based.

## Data Availability Statement

The whole genome sequencing data have been deposited to SRA at NCBI PRJNA428852.

**Author Contributions**

RH and YK conceived and designed the study. RH, IK, and KB-R performed experimental lab work. GH processed the sequencing raw data. RH, GH, EL, MS, and YK performed statistical analysis and interpretation of results. MP predicted the effect of candidate mutations on the 3D protein structure. RH, GH, EL, MS and YK wrote the manuscript. All authors read and approved the final manuscript.

## Conflict of Interest

The authors declare that the research was conducted in the absence of any commercial or financial relationships that could be construed as a potential conflict of interest.

## Supplementary Material

Supplementary Data sheet 1 Allele frequency estimates of all pools, for each of the 35k markers, for growth.

Supplementary Data sheet 2 Allele frequency estimates of all pools, for each of the 35k markers, for survival.

Supplementary Data sheet 3 QTL listing.

Supplementary Data sheet 4 The positions of mapped SNPs and indels.

Supplementary Data sheet 5 The phenotypes of the segregants.
