# Supplementary material for: Mapping Ethanol Tolerance in Budding Yeast Reveals High Genetic Variation in a Wild Isolate
Source: Front Genet. 2019 Nov 20;10:998. doi: 10.3389/fgene.2019.00998 (PMC6879558; doi:10.3389/fgene.2019.00998)
Supplement: Supplementary file 6 [file Presentation_1.pdf]

Supplementary Figures

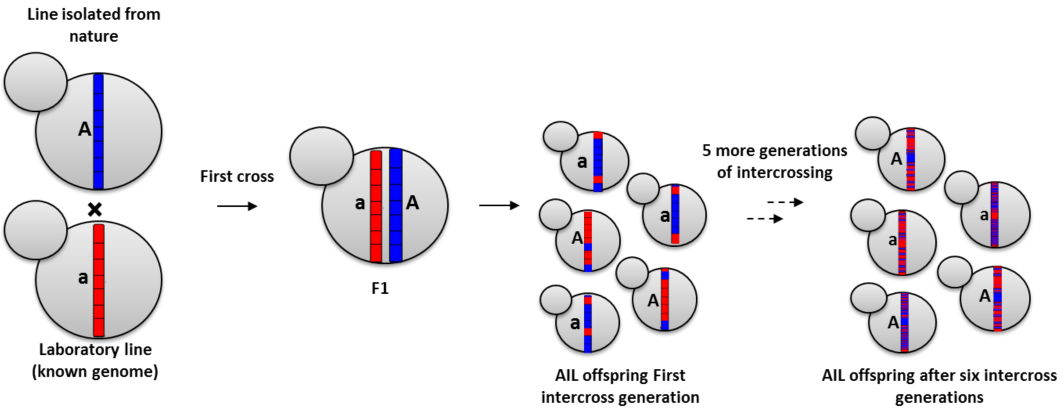

Supplementary Figure 1. The AIL scheme for fine mapping. A haploid strain isolated from nature (YE-531) (Ezov et al., 2006) and a laboratory haploid strain (S228c) were crossed to create an F1. The F1 went through EZ ascospore isolation procedure (Bahalul et al., 2010) followed by germination and intercrosses for 5 more generations, creating a mosaic genome. A/a, QTL alleles (Darvasi and Soller, 1995).

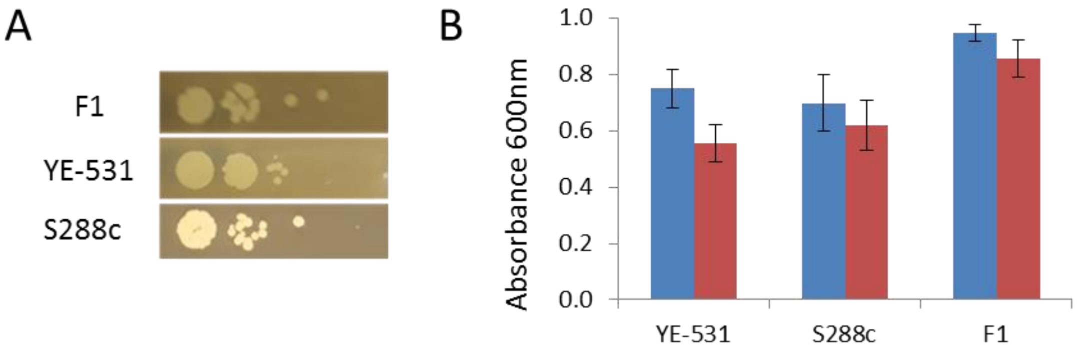

Supplementary Figure 2. Ethanol tolerance of parental lines**.** (A) Survival of parental lines and the F1 in the presence of ethanol. Cells from each strain were incubated in YPD medium in 19% (V/V) ethanol at 30°C for 5 hours, in anaerobic conditions. Each drop was seeded in spot assay at 1 to 100,000 fold dilution. The process was done in 4 biological repeats. (B) Growth ability of parental lines and the F1 in the presence of 9.5% (V/V) ethanol (Blue) and 10.8% (V/V) ethanol (red). Cells from each strain, in equal concentrations, were grown in YPD medium with ethanol content at 30°C for 22 hours, in anaerobic condition. Final 600nm absorbance was measured. The mean values and SD are presented.

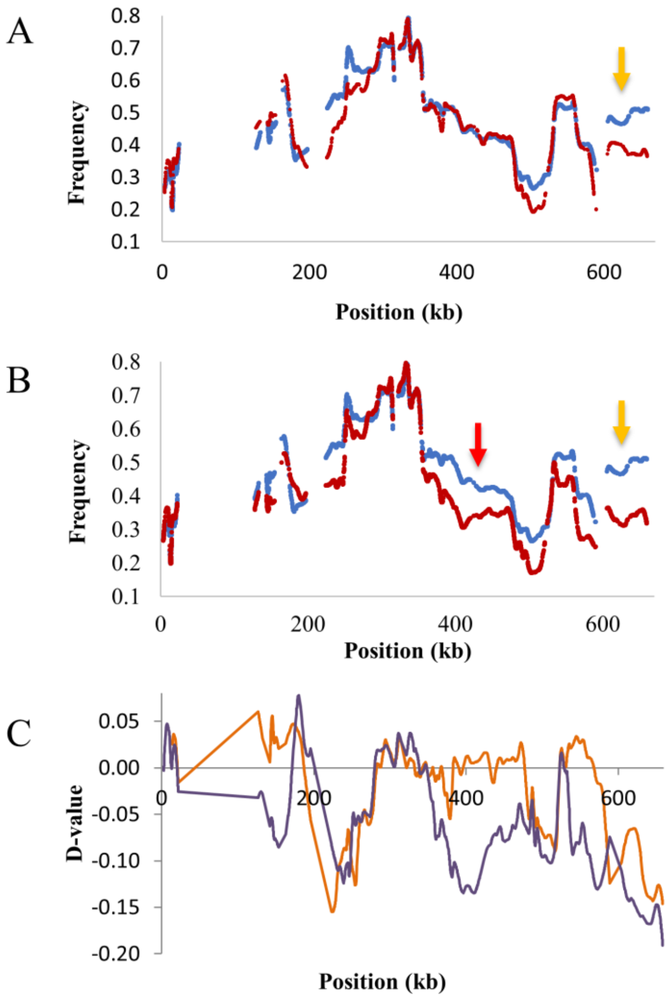

Supplementary Figure 3. Smoothed mean SNP Allele frequency and D-values on chromosome 11. Average frequency of YE-531 allele of the 3 replicates of a group, after LOESS smoothing. Blue: F6 (the same control groups were used for both traits); Red: upper tail; (A) Growth; (B) Survival. Some regions show large differences between the allele frequencies of tails and controls (arrows: Red arrow, significant region only for survival; orange arrows, significant region for both traits). (C) The allele frequency difference between the tail groups and the F6 (D-values). Orange: growth; Purple: survival.

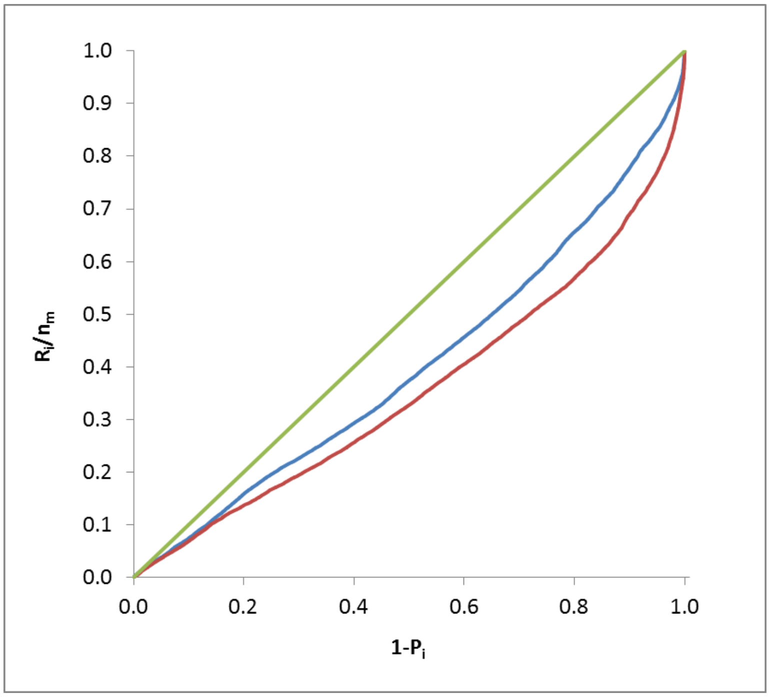

Supplementary Figure 4. Schweder-Spjotvoll plot (Spjotvoll, 1982) for P-values after LOESS smoothing. Values of 1-P_i were sorted in ascending order. Then R_i/n_M, the rank number of the ith marker divided by the total number of marker tests (n_M), was plotted against the sorted 1-P_i values. Green, expected 45° regression line, the expectation if all marker-tests were under the null-hypothesis; Blue and red, the actual results of Growth and Survival. The slopes of the curves are distinctly less than 45° in the first half of the chart (small 1-P_i = large P_i), reflecting a deficiency of true null-hypotheses in the dataset. On the other hand, the slopes of the curves are distinctly greater than 45° toward the right hand side of the chart (small P_i), reflecting a large number of rejected null-hypotheses (true, significant QTL effects). The greater concavity of the survival curve indicates a larger number of significant markers. As shown, the regression lines may be used to estimate the number of true null hypotheses (Spjotvoll, 1982; Lipkin et al., 2016).

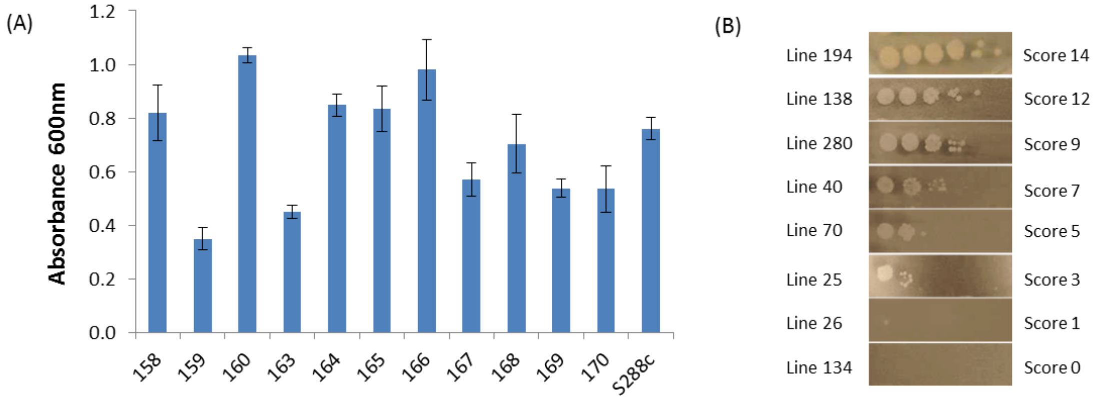

Supplementary Figure 5. An example of ethanol tolerance of the segregants. (A) Growth ability of 11 segregants (out of 300 tested) and the founder S288c in 9.5% (V/V) ethanol. Cells were grown in YPD medium with ethanol at 30°C for 20 hours, in anaerobic conditions. Final 600nm absorbance was measured. The mean values and SD are presented. (B) Survival test of 8 segregants (out of 300 tested) in the presence of 19% (V/V) ethanol for 5 hours. Cells in equal concentrations were incubated in YPD medium with ethanol at 30°C for 5 hours, in anaerobic conditions. Samples were seeded at 1 to 100,000-fold dilutions. Survival score (0-14) was assigned depending on the final dilution showing survival in the spot assay (see examples on the right).

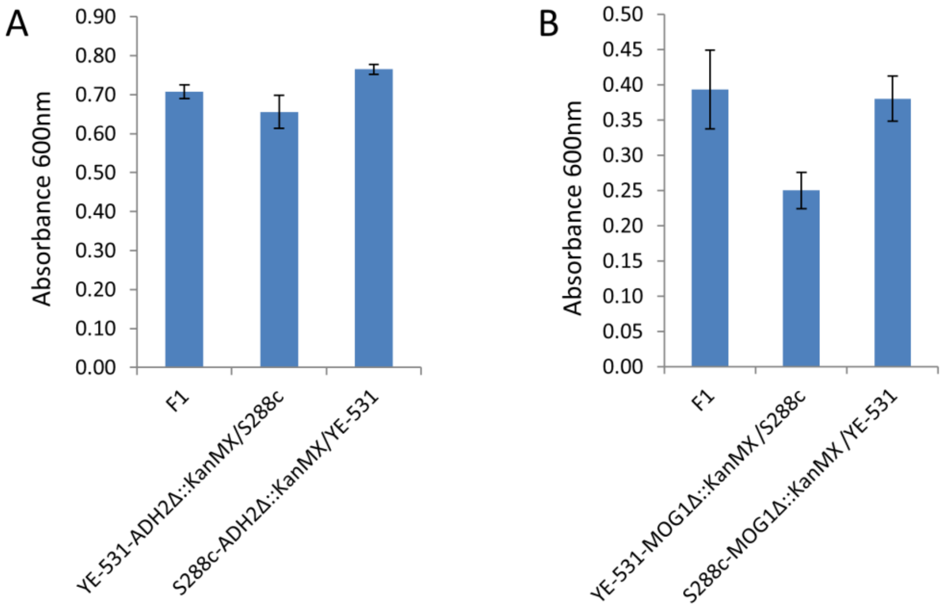

Supplementary Figure 6. Reciprocal hemizygosity analysis confirms the contribution of *ADH2* and *MOG1* to ethanol tolerance trait**.** Growth ability tests under ethanol stress (left, 10%; right, 11%) of F1 and *ADH2*/*MOG1* reciprocal hemizygous deletions. The mean values of 4 repeats in a single batch and the SD are presented. See tests for statistical significance, based on two batches, in Supplementary Table 9. (A) *ADH2* gene contributes to ethanol tolerance trait. The effect was small, as might be expected for effect of a single gene only, but in the correct direction and statistically significant (Supplementary Table 9). (B) *MOG1* gene contributes to ethanol tolerance trait. F1 hybrids were individually deleted for the *ADH2* and *MOG1* alleles. In both cases, the deletion of S288c-alleles in the background of the F1 strain resulted in a better growth ability than of the deletion of YE-531-alleles (YE-531-allele increase ethanol tolerance).

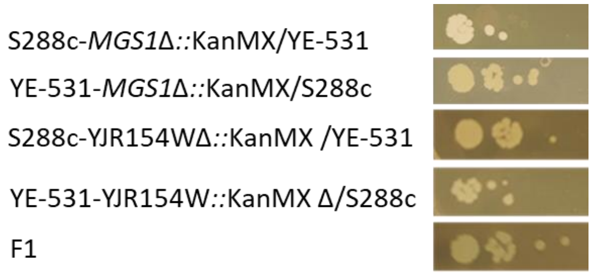

Supplementary Figure 7. Reciprocal hemizygosity analysis confirms the contribution of YJR154W and *MGS1* to ethanol survival trait. Survival ability tests of reciprocal hemizygous deletions, was tested under ethanol stress (19%(V/V). In the background of the F1 strain, the deletion of YE-531-allele (and expression of the S288c allele) resulted in a better survival ability than of the deletion of S288c allele for *MGS1* (and expression of the YE-531 allele). Thus, YE-531-allele increased ethanol tolerance). The opposite was obtained for YJR154W, as in this case YE-531-allele increased ethanol tolerance.

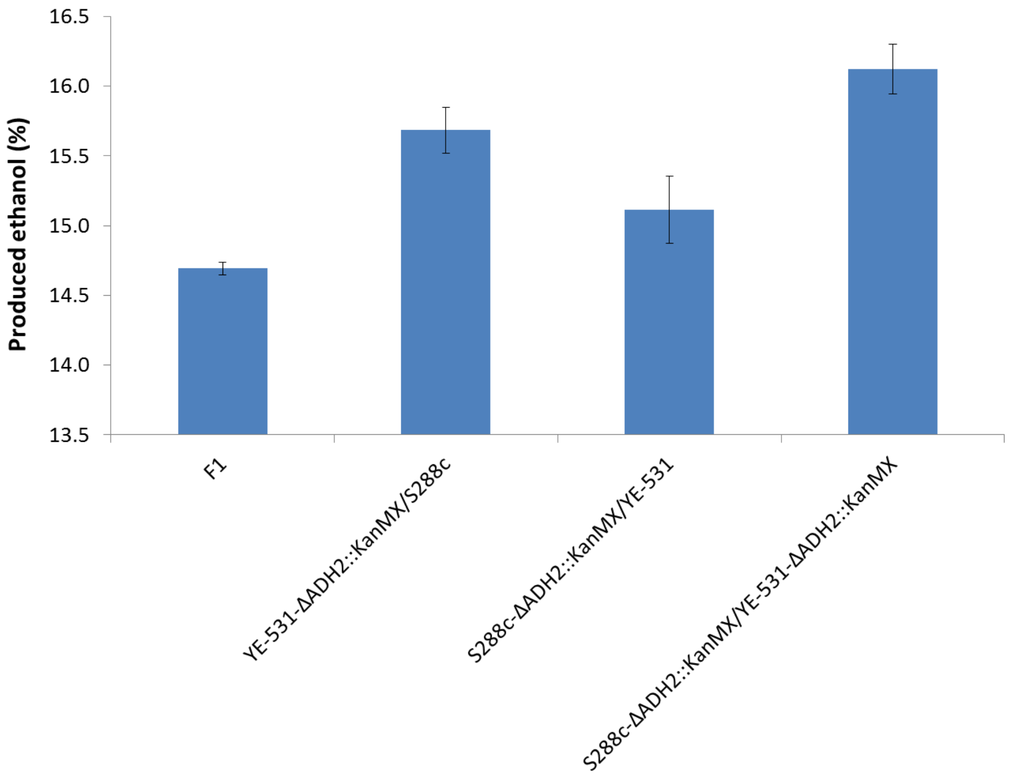

Supplementary Figure 8. Improved ethanol production by *ADH2* deletion strains in accordance to RHA. Shown is average ethanol production of the F1, *ADH2* reciprocal hemizygous deletions, and a double deletion strain in the F1 background, after 17 fermentation hours. Results are the average of 2 biological replicates (for each one 2 technical repeats). Fermentations were conducted using corn mash at 32.5°C and 160 rpm agitation, under anaerobic conditions, with initial cell concentration of 10^7 cells/ml.

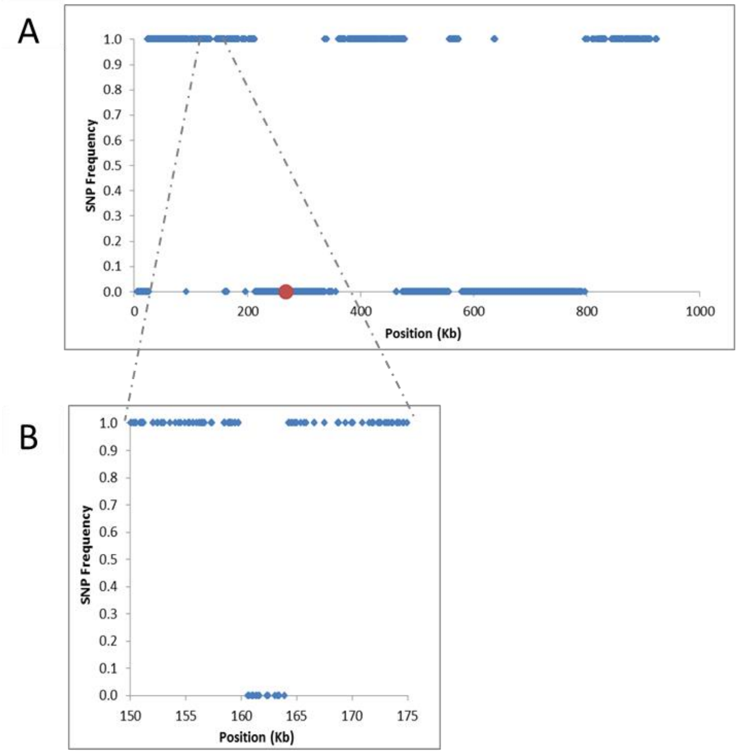

Supplementary Figure 9. Recombination block structure and frequency distribution of parental line alleles. (A) Origin of founder SNPs across chromosome 13 of Line-8, an individual F6 haploid segregant. Sequences were aligned to the reference S288c genome sequence. Thus, SNP frequencies 0 or 1 indicate an S288c or YE-531 allele, respectively. Red circle, centromere; (B) Zoom-in on a small haplotype block of size 4,554 bp.

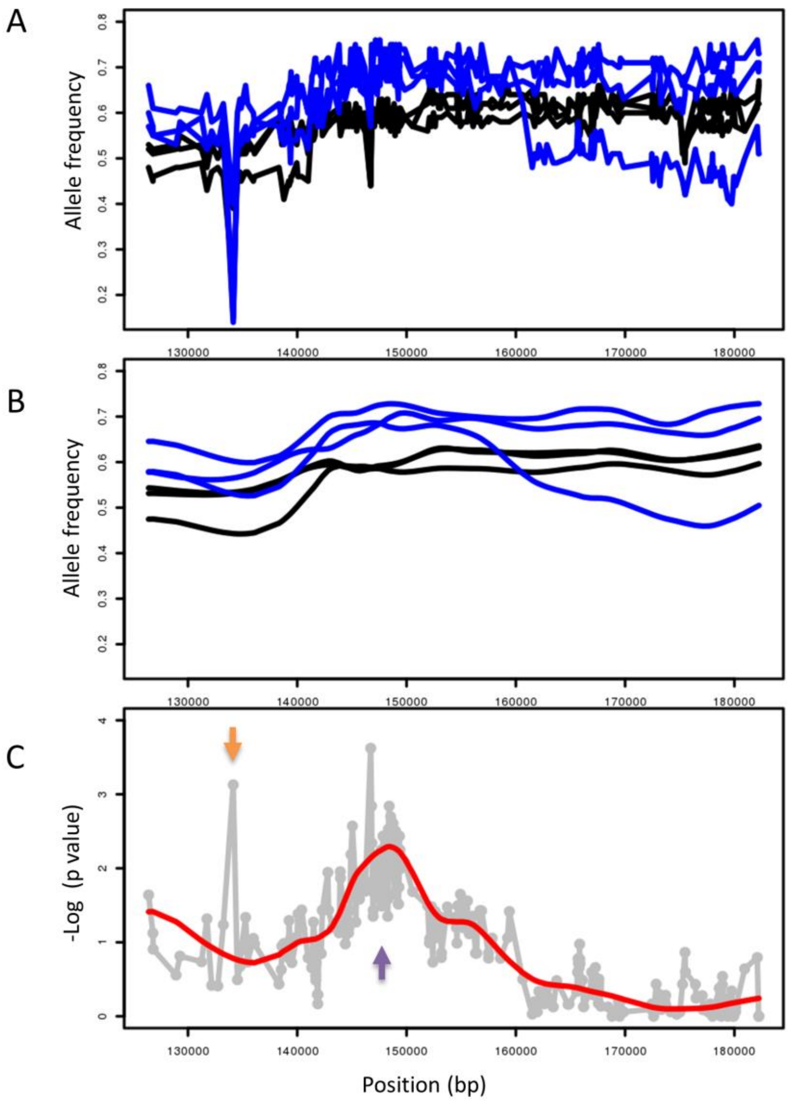

Supplementary Figure 10. Example for LOESS smoothing effect on allele frequencies (chromosome 8 for growth trait). Blue, resistant pools; Black, Control pools. (A) Raw allele frequencies show a large background noise. (B) Improved smoothed frequencies consistency due to LOESS smoothing. (C) Results significance is affected by LOESS smoothing. Orange arrow, false positives are reduced; Purple arrow, clarification of a peak shape after LOESS**.**

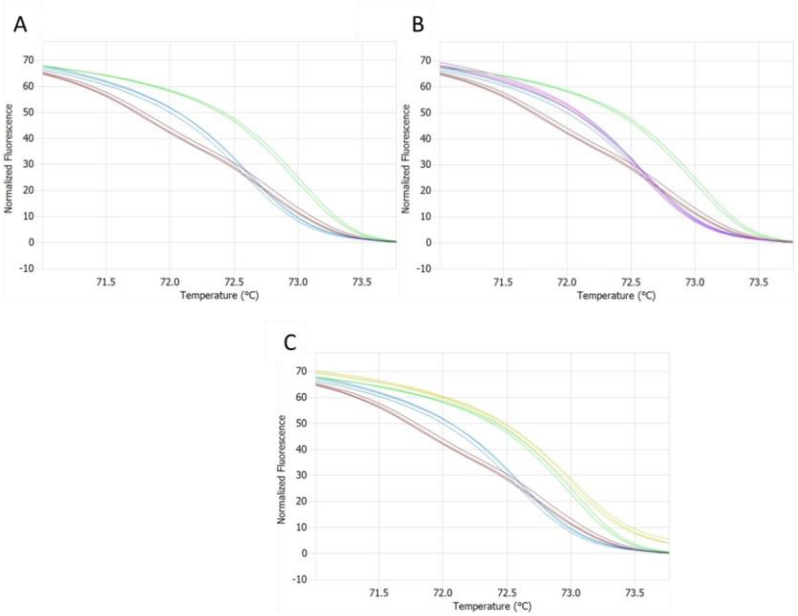

Supplementary Figure 11. Individual frequency estimates of a tested SNP by real time PCR coupled to High Resolution Melting (HRM) genotyping. The charts are focused on the T_m area for a SNP located in the gene UPT20 on chromosome 2 position 228,251 (examined for 30 individuals which composed one of the survival upper tail group and 30 random segregants of the entire F6 population) (A) HRM results for S288c (blue), YE-531 (green) and a mix of both (brown). (B) HRM results for S288c (blue), YE-531 (green) mix of both of them (brown) and segregant YE-159 having the allele of S288c (pink). (C) HRM results for S288c (blue), YE-531 (green) mix of both (brown) and one of the segregant YE-270 having the allele of YE-531 (yellow).

Supplementary Tables

Supplementary Table 1 full sequence identifiers of the genome sequences and the strains description that were used for the tree building in fig. 1

Name	Full sequence identifiers	Description
CLIB324	CLIB324_WashU_2011_AEWM01000000	Vietnamese bakery isolate
D273-10B	D273-10B_Stanford_2014_JRIY00000000	Laboratory strain
DBVPG6044	DBVPG6044_Stanford_2014_JRIG00000000	Derivative of West African bili wine isolate
EC9-8	EC9-8_ASinica_2011_AGSJ01000000	Haploid derivative of Israeli canyon isolate
FL100	FL100_Stanford_2014_JRIT00000000	Laboratory strain
K11	K11_Stanford_2014_JRIJ00000000	Japanese sake yeast
L1528	L1528_Stanford_2014_JRIK00000000	Chilean red wine isolate
RedStar	RedStar_Stanford_2014_JRIL00000000	Commercial baking yeast
RM11-1A	RM11-1A_SGD_2015_JRIP00000000	Derivative of California Zinfandel vineyard
S288C	S288C_reference_sequence_R64-2-1	Laboratory strain
T73	T73_WashU_2011_AFDF01000000	Spanish red wine strain
Vin13	Vin13_AWRI_2010_ADXC01000000	South African white wine
YPS128	YPS128_Stanford_2014_JRID00000000	Pennsylvania woodland isolate
YS9	YS9_Stanford_2014_JRIB00000000	Singaporean commercial baking strain

Supplementary Table 2. Distribution of YE-531 SNP allele frequencies in the F6 population

Frequency	N	Proportion
0.0-0.1	292	0.008
>0.1-0.2	1,408	0.040
>0.2-0.3	3,726	0.106
>0.3-0.4	6,375	0.181
>0.4-0.5	6,753	0.192
>0.5-0.6	5,894	0.168
>0.6-0.7	5,527	0.157
>0.7-0.8	3,968	0.113
>0.8-0.9	1,134	0.032
>0.9-1.0	57	0.002
F≤0.5	18,554	0.528
Sum	35,134	1.000

Frequency, range of frequencies; N, number of markers having frequency of the YE-531 allele in this range; Proportion, proportion of the markers in this range among all 35,134 markers; F≤0.5, proportion of all markers having this frequency among all markers.

Supplementary Table 3. Distribution of D-values before and after LOESS

	**Before LOESS**				**After LOESS**			
	**Growth**		**Survival**		**Growth**		**Survival**	
	**N**	Proportion	**N**	Proportion	**N**	Proportion	**N**	Proportion
**>-0.4 - -0.3**	1	0.00003	2	0.00006	0	0.00000	0	0.00000
**>-0.3 - -0.2**	41	0.00114	48	0.00134	2	0.00006	39	0.00111
**>-0.2 - -0.1**	1,577	0.04401	2,550	0.07116	727	0.02069	2,640	0.07539
**>-0.1 - 0.0**	16,127	0.45005	16,030	0.44734	17,649	0.50233	14,360	0.41006
**>0.0 - 0.1**	17,063	0.47617	14,340	0.40018	15,481	0.44063	15,923	0.45470
**>0.1 - 0.2**	1,001	0.02793	2,798	0.07808	1,272	0.03620	2,056	0.05871
**>0.2 - 0.3**	14	0.00039	62	0.00173	1	0.00003	1	0.00003
**>0.3 - 0.4**	7	0.00020	2	0.00006	1	0.00003		0.00000
**>0.4 - 0.5**	1	0.00003	1	0.00003	1	0.00003		0.00000
**>0.5 - 0.6**	1	0.00003		0.00000		0.00000		0.00000
**>0.6 - 0.7**	1	0.00003	1	0.00003		0.00000		0.00000
**D≤0**	17,746	0.49523	18,630	0.51990	18,378	0.52308	17,039	0.48656
**D>0**	18,088	0.50477	17,204	0.48010	16,756	0.47692	17,980	0.51344
**Total**	35,834	1.00000	35,834	1.00000	35,134	1.00000	35,019	1.00000

[1]N, number of markers having D-values in this range; Proportion, proportion of the markers in this range among all markers.

[2]D-value was defined as the allele frequency difference between the mean of the three tail groups and the mean of the three control groups for each trait. After LOESS, 94% of D-values were in the range -0.1 to 0.1 for growth, and 86% were in this range for survival.

Supplementary Table 4. Distribution of marker P-values

	**Proportion**	
**P-value**	**Growth**	**Survival**
≤0.1	0.223	0.310
>0.1 - 0.2	0.121	0.122
>0.2 - 0.3	0.111	0.084
>0.3 - 0.4	0.089	0.079
>0.4 - 0.5	0.082	0.078
>0.5 - 0.6	0.081	0.071
>0.6 - 0.7	0.067	0.062
>0.7 - 0.8	0.068	0.057
>0.8 - 0.9	0.084	0.068
>0.9 - 1.0	0.074	0.068
Total	35,134	35,019

[1]Presented are the nominal P-values. To define QTLs we used FDR cutoff and chose the critical threshold P-values accordingly.

[2]Proportion, proportion of the markers in this range among all markers.

Supplementary Table 5. Distribution of QTLs and positive alleles across the genome

(A) Growth

Chr	N	Proportion	S288c	YE-531
1	0	-	-	-
2	3	0.0588	1	2
3	2	0.0392	2	0
4	3	0.0588	-	3
5	0	-	-	-
6	1	0.0196	0	1
7	6	0.1176	2	4
8	4	0.0784	2	2
9	2	0.0392	-	2
10	3	0.0588	1	2
11	5	0.0980	5	-
12	1	0.0196	1	-
13	4	0.0784	1	3
14	4	0.0784	2	2
15	5	0.0980	3	2
16	8	0.1569	4	4
Sum	51	1.0000	24	27

(B) Survival

Chr	N	Proportion	S288c	YE-531
1	0	0.0000	0	0
2	8	0.0833	3	5
3	4	0.0417	0	4
4	9	0.0938	2	7
5	8	0.0833	8	0
6	1	0.0104	0	1
7	9	0.0938	6	3
8	7	0.0729	5	2
9	4	0.0417	1	3
10	5	0.0521	4	1
11	8	0.0833	8	0
12	7	0.0729	5	2
13	6	0.0625	0	6
14	6	0.0625	1	5
15	2	0.0208	1	1
16	12	0.1250	3	9
Sum	96	1	49	47

Proportion is proportion of QTL on given chromosome out of all mapped QTL by trait.

Supplementary Table 6. Overall distribution by traits of significant SNPs and indels among QTLs, ORFs and regulatory sites

**Content**		**Growth**	**Survival**
**QTL**	SNPs	3,592	7,204
	Indels	351	732
	ORFs	311	614
	Regulatory sites	81	145
**ORF**	Indels	57	112
	Syn SNPs	1,073	2,055
	Non-Syn SNPs	616	1,167
**Regulatory sites**	Indels	19	23
	SNPs	62	122

Syn SNPs, SNPs resulting in synonymous nucleotide substitutions; Non-Syn SNPs, SNPs resulting in nonsynonymous substitutions. Only SNPs used for QTL mapping are included.

Supplementary Table 7. Distribution of QTL according to number of ORFs within the QTL

ORF/QTL	Survival		Growth	
	N	Proportion	N	Proportion
0	3	0.031	2	0.039
1	10	0.104	5	0.098
2	5	0.052	3	0.059
3	9	0.094	4	0.078
4	11	0.115	6	0.118
5	9	0.094	6	0.118
6-10	33	0.104	22	0.157
11-20	13	0.094	1	0.078
>20	3	0.042	2	0.098
Total	96	1.000	51	1.000

Supplementary Table 8. QTL allele effect (δ) and contribution to the phenotypic variance (cP)

(A) Growth (OD units at 600 nm)

Chr	QTL	δ	cP	Chr	QTL	δ	cP	Chr	QTL	δ	cP
2	1	0.034	0.020	8	18	0.044	0.031	14	35	0.019	0.006
2	2	0.021	0.007	8	19	0.015	0.005	14	36	0.019	0.003
2	3	0.021	0.005	9	20	0.015	0.005	14	37	0.013	0.003
3	4	0.012	0.003	9	21	0.012	0.003	14	38	0.022	0.009
3	5	0.014	0.004	10	22	0.018	0.006	15	39	0.017	0.007
4	6	0.013	0.004	10	23	0.021	0.007	15	40	0.015	0.004
4	7	0.015	0.004	10	24	0.014	0.003	15	41	0.013	0.004
4	8	0.021	0.008	11	25	0.020	0.009	15	42	0.014	0.004
6	9	0.027	0.016	11	26	0.017	0.006	15	43	0.019	0.007
7	10	0.015	0.005	11	27	0.015	0.004	16	44	0.019	0.008
7	11	0.025	0.010	11	28	0.020	0.008	16	45	0.015	0.005
7	12	0.017	0.005	11	29	0.019	0.008	16	46	0.013	0.004
7	13	0.014	0.004	12	30	0.014	0.003	16	47	0.012	0.003
7	14	0.019	0.008	13	31	0.020	0.009	16	48	0.013	0.004
7	15	0.015	0.005	13	32	0.017	0.006	16	49	0.016	0.004
8	16	0.019	0.007	13	33	0.016	0.005	16	50	0.013	0.003
8	17	0.037	0.025	13	34	0.020	0.009	16	51	0.014	0.004
								Avg		0.018	0.007
								Total			0.347

(B) Survival (survival trait units)

Chr	QTL	δ	cP	Chr	QTL	δ	cP	Chr	QTL	δ	cP
2	1	0.244	0.003	7	33	0.334	0.006	12	65	0.309	0.006
2	2	0.584	0.017	7	34	0.561	0.010	12	66	0.251	0.004
2	3	0.480	0.006	7	35	0.279	0.005	12	67	0.479	0.009
2	4	0.436	0.006	7	36	0.337	0.007	12	68	0.353	0.006
2	5	0.460	0.009	7	37	0.269	0.005	12	69	0.328	0.005
2	6	0.489	0.010	7	38	0.320	0.006	12	70	0.373	0.006
2	7	0.405	0.007	7	39	0.286	0.005	13	71	0.287	0.005
2	8	0.550	0.012	8	40	0.423	0.011	13	72	0.415	0.010
3	9	0.337	0.006	8	41	0.452	0.013	13	73	0.258	0.004
3	10	0.335	0.005	8	42	0.296	0.006	13	74	0.254	0.004
3	11	0.377	0.009	8	43	0.794	0.033	13	75	0.413	0.009
3	12	0.455	0.012	8	44	0.376	0.007	13	76	0.253	0.004
4	13	0.284	0.005	8	45	0.240	0.004	14	77	0.576	0.006
4	14	0.231	0.003	8	46	0.246	0.004	14	78	0.693	0.013
4	15	0.316	0.006	9	47	0.249	0.004	14	79	0.265	0.004
4	16	0.364	0.009	9	48	0.234	0.003	14	80	0.250	0.004
4	17	0.274	0.005	9	49	0.303	0.006	14	81	0.313	0.006
4	18	0.294	0.005	9	50	0.412	0.011	14	82	0.207	0.003
4	19	0.294	0.005	10	51	0.415	0.011	15	83	0.308	0.005
4	20	0.274	0.004	10	52	0.247	0.004	15	84	0.311	0.006
4	21	0.550	0.017	10	53	0.297	0.006	16	85	0.277	0.005
5	22	0.301	0.005	10	54	0.309	0.006	16	86	0.403	0.010
5	23	0.358	0.008	10	55	0.418	0.008	16	87	0.388	0.010
5	24	0.347	0.008	11	56	0.341	0.008	16	88	0.336	0.005
5	25	0.372	0.009	11	57	0.391	0.010	16	89	0.301	0.006
5	26	0.281	0.005	11	58	0.299	0.005	16	90	0.426	0.012
5	27	0.281	0.005	11	59	0.387	0.008	16	91	0.356	0.008
5	28	0.285	0.005	11	60	0.258	0.004	16	92	0.303	0.006
5	29	0.351	0.007	11	61	0.260	0.004	16	93	0.424	0.011
6	30	0.564	0.020	11	62	0.394	0.010	16	94	0.267	0.005
7	31	0.264	0.004	11	63	0.525	0.018	16	95	0.551	0.018
7	32	0.274	0.005	12	64	0.441	0.011	16	96	0.266	0.004
	Avg		0.357	0.008							
	Total			0.720							

δ, allele effect; cP, the contribution of the QTL to the phenotypic variance.

Supplementary Table 9. Comparison of QTL and RHA effects

**Gene**	**Standard name**	**Trait**	**Favor QTL allele**	**RHA Allele effect**	**Favor RHA allele**	**P**	**Sign**	
				S288c-Δ::kanMX/ YE-531	YE-531-Δ::kanMX/ S288c			
ADH2	YMR303C	G	YE-531	0.594	0.500	YE-531	0.010	+
MOG1	YJR074W	G	YE-531	0.521	0.433	YE-531	0.0020	+
None	YJR154W	G	YE-531	0.649	0.636	YE-531	0.38	+
RTG2	YGL252C	G	S288c	0.819	0.830	S288c	0.42	+
MGS1	YNL218W	S	S288c	1.073	1.135	S288c	0.0060	+
ZRT1	YGL255W	S	S288c	1.250	1.313	S288c	0.40	+
NTH2	YBR001C	S	S288c	0.986	0.986	EQUAL	0.19	NI
MMP1	YLL061W	S	S288c	1.010	1.063	S288c	0.18	+
None	YJR154W	S	YE-531	0.992	0.767	YE-531	0	+
ADH2	YMR303C	S	YE-531	1.104	1.083	YE-531	0.20	+

G, growth; S, survival; RHA Allele effect, average effect; P, CWER p-value by ANOVA (growth trait) or nonparametric ANCOVA (survival). Sign: +, QTL mapping and RHA agree on favorable allele; -, QTL and RHA disagree; EQUAL, the two alleles have exactly the same effect on RHA; NI, comparison is not informative for sign test.

Supplementary Table 10. Recombination block size distribution across the entire genome of one random F6 individual, Line-8

**haplotype size (Kb)**	**N**	**%**
0.01-0.10	3	1.43
0.1-1.00	22	10.48
1-5	48	22.86
5-10	20	9.52
10-100	77	36.67
100-1,000	40	19.05
**Total**	**210**	
**Median**	**16.1 Kb**	

Supplementary Table 11. Sequencing coverage of analyzed pool replicates

Sample	Coverage of reads
Control -replicate1	3,180
Control -replicate2	2,217
Control -replicate3	1,842
Growth upper tail replicate1	968
Growth upper tail replicate2	1,009
Growth upper tail replicate3	946
Survival upper tail replicate1	1,013
Survival upper tail replicate2	951
Survival upper tail replicate3	1,019
Random F6 haploid individual	989
S288c	705
YE-531	1,385

AIL F6 population served as the control

Supplementary Table 12. Primers used for HRM analyses

Name	Sequence 5' to 3'	Amplicon length
UPT20-F	TCTTTCAAGATTTTCAGCAGAAGCA	114
UPT20-R	AAAAGTTAGAGGGAGATGATGAGCA	
PRP28-F	ACCAGGATGTCACAACCCTC	105
PRP28-R	TCATAGTTGCTCTCTTTGGACCAT	

Supplementary Table 13. Comparison of HRM and deep sequencing frequency estimates

				HRM				DS[1]
SNP	Chr	bp	Pool	SS	YY	SY	Y allele frequency	Y allele frequency
1	4	949,385	Growth	11	7	12	0.433	0.430
			Population	6	9	15	0.550	0.670
2	2	228,251	Survival	12	12	6	0.500	0.550
			Population	2	17	11	0.750	0.730

bp, position in bp; Pool, Source of segregants; S, S288c allele; Y, YE-531 allele; HRM, Y-allele frequency estimate obtained by HRM; DS, Y-allele frequency estimate obtained by deep sequencing.

[1]DS frequencies directly obtained after alignment to S288c reference genome, before smoothing by LOESS.

Supplementary Table 14. Oligonucleotides utilized as specific deletion primers for “cassettes" constructions for transformation (for RHA)

Name	Sequence 5’ to 3’
YBR001C-F	GCGCTTGTAGGAACTGTT
YBR001C-R	GACGATTTAGAGTAAGGTC
YGL255W-F	GCATTAGCTCGATGACTTAG
YGL255W-R	CAGTCTCGGACAATAAATACGC
YJR154W-F	CATGTGTTGATAGCAGGTGACG
YJR154W-R	CTATTCTAGTACTTCCCTGCTG
YLL061W-F	GATGCCAGGAAATAAATGCG
YLL061W-R	GAATGATATTCTAGGCCCTG
YNL218W-F	GACATTCAATCATCGGTTGC
YNL218W-R	CAATGCCGCGTCTACAATTC
YGL252C-F	GAATGCCGAGATAGGATAAC
YGL252C-R	CACCTTCTTGTTGTTCAAAC
YMR303C-F	GCTATAGCATGCCTATCAC
YMR303C-R	TCACTCGTGCTAGCAAAC
YJR074W-F	GGACTGACTCCTTCATCGC
YJR074W-R	CACTTTCTTCGCTGCTGG

Deletion KanMX "cassettes" were constructed using PCR reactions for the BY4741 deletion strains, with primers designed (50-250) bp upstream and downstream each candidate ORF. The reaction was made to extend the specific ORF homology to enable mitotic recombination of the gene disruption cassette. F, forward primers; R, reverse primers.

Supplementary Table 15. Specific primers that were used for deletion strain confirmation (for RHA)

Name	Sequence 5’ to 3’
KanMX+ (used for all tests)	GCGCTTGTAGGAACTGTT
YBR001C-CheckR	TCAAGTTGTGTAAAGGCTC
YGL255W- CheckR	CAAGTGGTACCAGAATACG
YJR154W-CheckR	GGTGTGTCTGATACTCCTGC
YLL061W-CheckR	GCACGTCCAGGTTCTGTGAC
YNL218W- CheckR	AGATAATCAAGGATCCACC
YGL252C-CheckR	GTTTAAGCACCGATGATACC
YMR303C- CheckR	GAGACGATTCAGAGGAGCA
YJR074W- CheckR	CGAATATCATCAACCTCCTG

Selected colonies after “cassette” transformation were further verified by PCR for correct replacement of the gene with KanMX. PCR was performed using internal and external primers to the cassette. The internal forward primer was KanMX+, for all tests; the external reverse primer was CheckR, which is specific to each site. Appearance of PCR products of the expected size proved the correct replacement of the gene with KanMX.

Supplementary Text

1. Control group

SDP is typically implemented by selection of alternative tail groups from the overall phenotypic distribution of the mapping population. For survival, selection of the tolerance tail is achieved simply by exposing the population to the selective agent (ethanol in our case), and retaining survivors. Selection of the susceptible tail for survival, however, is technically complex since individuals that do not survive cannot be used to produce progeny; and non-survivors may also include numerous genes affecting normal cell function. Therefore, as suggested by Lebowitz et al., 1987 (Lebowitz et al., 1987), we took aliquots from the unselected AIL F6 population (more than 3.5 x 10^5 segregants/cc), to serve as the control group.

2. Preparation of DNA samples

Genomic DNA from founder strains and pools was extracted with MasterPure yeast DNA purification kit (Epicenter, Madison, WI), according to the manufacturer's instructions.

We performed whole genome deep sequencing of three DNA pools for each tail of each trait (total 6 tail pools; 30 segregants per pool). Thus, for each marker, three independent estimates of allele frequency (each based on pools of 30 segregants) were obtained for each trait.

For DNA extraction, for each of the 6 tail pools 3 subgroups were formed, each composed of 10 segregants. Based on OD600 nm absorption, an equal number of cells was taken from each segregant. Cells of the ten segregants of the subgroup were pooled together, and DNA was extracted. After extraction, equal DNA amounts of the 3 subgroups were pooled, representing the 30 individual segregants in each pool. For each of the three controls, DNA was extracted from an aliquot taken from an unselected AIL-F6 culture with cell density of 10 OD600 nm.

3. Confirmation of pool frequency estimates

Pool frequency estimates of two SNPs: on chromosome 2 position 228,251, and on chromosome 4 position 949,385, were confirmed by real time PCR coupled to high resolution melting (HRM) genotyping (Sean Taylor, 2010) (Supplementary Figure 11). Thirty individuals randomly chosen from the general AIL F6 population were genotyped for both SNPs, along with two groups of 30 individuals from each of the tail pools of growth and survival. DNA of the tested strains was extracted for each individual and diluted into equal concentration of 100ng/ml. Amplification was performed using Eco real-Time PCR system (Illumina) in 3 replicates for each strain. Amplification reaction consisted of 1µl DNA, 0.2µl of 10µM of forward and reverse primers (Supplementary Table 12), 5 µl reaction mix (FastSYBER green master mix, Applied Biosystems), 3.6 µl ddH_2O. Analyses were done by Eco version 4 software. SNP frequency was calculated for each group and compared to that obtained by deep f

The correlation between HRM and SDP sequencing for these markers was high (r = 0.891, Supplementary Table 13).

4. **Variance estimation**

As the three replicate pools in each tail consisted of 30 diploid segregants each, the number of chromosomes per tail pool was 60, and the expected variance among replicate pools would be pq/60, where p is the frequency of the YE-531 allele and q = 1-p. Taking a maximum value of pq = 0.25, we expect variance among replicate pools = 0.0042. In reality, for most of the SNPs pq < 0.25 (Supplementary Table 2).

5. **Schweder-Spjotvoll plot**

Values of 1-Pi. after LOESS smoothing were sorted in ascending order for each trait. Then, R_i/n_M, the rank number of the ith marker divided by the total number of marker tests (n_M), was plotted against the sorted 1-Pi. values.

6. **An example for allele substitution effect calculation**

The frequencies of the YE-531 allele in marker ref|NC_001134| was 0.696 and 0.594 in the pools of the selected survival tail and the entire F6 population, respectively; the estimated frequencies of the homozygotes in the two groups were calculated as the squares of these values, 0.485 and 0.353, respectively. The frequencies of the S228c allele were calculated as 1 minus 0.696 and 1 minus 0.594, respectively, and homozygotes were estimated accordingly. The mean growth frequency values were 0.923 and 0.691 in the selected and the entire F6 populations. The weighted means of the YE-531 homozygotes across both groups was thus calculated as (0.485*0.923 + 0.353*0.691) / (0.485 + 0.353) = 0.825, and the weighted mean of the S228c allele was calculated similarly; the difference between the two homozygote groups was calculated as d = 0.825 - 0.774 = 0.051; the selection intensity was $i_{p/2}$ = 1.343, thus δ = d/1.343 = 0.028.

7. **Reciprocal Hemizygosity Analysis**

For RHA (Steinmetz et al., 2002), deletions were made in the S288c and YE-531 haploid backgrounds, using the strategy of Yeast Deletion Project (http://www-sequence.stanford.edu/group/yeast_deletion_project/project_desc.html), based on the deletion strain collection (EUROSCARF). Deletion KanMX "cassettes" were constructed using PCR reactions for the BY4741 deletion strains, with primers designed 50-250 bp upstream and downstream of each candidate ORF (Supplementary Table 14). Selection for transformants was made on agar plates with G418 (Geneticin). Selected colonies were further verified by PCR (Supplementary Table 15). Reciprocal strains were generated by crossing the deletion parental with the other parental strain.

To determine whether one allele is advantageous over the other in the RHA tests, we tested growth and survival of the two reciprocal deletion strains under ethanol stress, as in the second stage of the pool construction. We tested 6 genes for a single trait only (growth or survival), and 2 genes for both traits, making a total of 10 gene x trait tests. Each deletion strain was tested in 2 - 5 "batches", each batch consisting of four replicates. For growth, the performance of the strain under 9.5-11 % ethanol was taken as the mean OD600 nm across the replicates and batches. For survival, each replicate was graded under 18% or 19% ethanol. All candidate genes were first tested under the ethanol concentrations

originally used (9.5% for growth and 19% for survival) to evaluate the F6 phenotypic abilities. Since only a single candidate gene was tested in the F1 background, unlike the tests performed in the F6, in some cases several more batches under slightly higher/lower ethanol concentrations were performed to detect differences between alternative alleles. The performance of the deletion strain was taken as the sum of grades across replicates and batches. Grade for a replicate was assigned as follows. Each replicate was tested at five 10-fold dilutions, giving a total of 6 dilutions (including 0 dilution); and each dilution was scored 0 (no growth) to 3 (full growth) by a visual inspection. The grade for the replicate was the simple sum of the dilution scores.

For example, Gene YNL218W deletion strain S288c-Δ::kanMX/YE-531 was graded in four batches. For Batch 1, Replicate 1, results were: Dilution 0, score 2; Dilution 1, score 2; Dilution 2, score 1; Dilution 3, score 1; Dilution 4 and 5, score 0. Total score: 6. Replicates 2, 3 and 4 had total scores 5, 7, 7, respectively, giving the grade 25 for Batch 1. In the same way, we obtained scores, 19, 19, 40 for Batches 2, 3, 4, for the grade 103 for S288c-Δ::kanMX/YE-531 deletion of the gene YNL218W. The average value was then calculated as the grade divided by the number of observations (103/96).

A two-way ANOVA (R aov) with batch and allele as main effects was used to test the null hypothesis of equal growth of the alternative deletion strains. A Non-parametric ANCOVA (Young and Bowman, 1995) test was used to test the null hypothesis of equal slopes obtained from regressing survival scores on serial dilutions.

For the significance of the overall hypothesis that effects on ethanol tolerance of alternative alleles at single genes as obtained by QTL mapping procedures, are validated by the RHA analyses, we used a nonparametric sign test to test the null hypothesis that the favorable allele identified by QTL mapping and the favorable allele identified by RHA are independent. To do this we compared the favorable allele by QTL mapping and favorable allele by RHA for each of 10 gene x trait combinations. Results of all three analyses are shown in Supplementary Table 9.

8. Ethanol production assay

Incubation of 200 ml final volume, of 10^7 cells/ml yeast cells suspended in ground degraded corn prepared following (Bothast and Schlicher, 2005), was in 250 ml Erlenmeyer flasks at 32.5°C under constant agitation (160rpm). After 17 hr of incubation, ethanol levels of the sample supernatant (after centrifugation at 3000 g x 2 min to remove solids) and of reference ethanol standards (European Reference Materials) were quantified using Thermo Scientific Trace 1300 gas chromatograph with flame ionization detector. Glucose levels were quantified using glucose (HK) assay kit (Sigma Aldrich) initially and after 17 hr.

9. Recombination blocks in a single randomly chosen F6 individual

The mapping based on pool analysis uncovered relatively narrow QTLs that we attribute to multiple recombinations accumulated during the 6 AIL generations. However, the mapped QTLs obtained by pool analysis do not represent the distribution of the size of recombination blocks of the individual segregants formed by AIL. To evaluate this, a single

F6 haploid segregant, Line-8, was sequenced (Supplementary Figure 9). A total of 210 blocks were found across this individual genome, with a median length of about 16.1 Kb. More than 30% of the blocks were smaller than 5 Kb (Supplementary Table 10). These results are in accord with the obtained narrow QTL. As expected, the narrow width of Line-8 blocks, confirms the efficacy of the AIL design in generating narrow haplotype blocks that increase mapping resolution, and supports the use of a 1 log drop interval to define QTL boundaries.

References

Bahalul, M., Kaneti, G., and Kashi, Y. (2010). Ether-zymolyase ascospore isolation procedure: an efficient protocol for ascospores isolation in Saccharomyces cerevisiae yeast. *Yeast* 27(12)**,** 999-1003. doi: 10.1002/yea.1808.

Bothast, R.J., and Schlicher, M.A. (2005). Biotechnological processes for conversion of corn into ethanol. *Appl Microbiol Biotechnol* 67(1)**,** 19-25. doi: 10.1007/s00253-004-1819-8.

Darvasi, A., and Soller, M. (1995). Advanced intercross lines, an experimental population for fine genetic mapping. *Genetics* 141(3)**,** 1199-1207.

Ezov, T.K., Boger-Nadjar, E., Frenkel, Z., Katsperovski, I., Kemeny, S., Nevo, E., et al. (2006). Molecular-genetic biodiversity in a natural population of the yeast Saccharomyces cerevisiae from "Evolution Canyon": microsatellite polymorphism, ploidy and controversial sexual status. *Genetics* 174(3)**,** 1455-1468. doi: 10.1534/genetics.106.062745.

Lebowitz, R.J., Soller, M., and Beckmann, J.S. (1987). Trait-based analyses for the detection of linkage between marker loci and quantitative trait loci in crosses between inbred lines. *Theoretical and Applied Genetics* 73(4)**,** 556-562. doi: 10.1007/bf00289194.

Lipkin, E., Strillacci, M.G., Eitam, H., Yishay, M., Schiavini, F., Soller, M., et al. (2016). The Use of Kosher Phenotyping for Mapping QTL Affecting Susceptibility to Bovine Respiratory Disease. *PLoS ONE* 11(4)**,** e0153423. doi: 10.1371/journal.pone.0153423.

Sean Taylor, R.S., Richard Kurts, Carl Fisher, Viresh Patel and Frank Bizouarn (2010). "A Practical Guide To High Resolution Melt Analysis Genotyping", in: *Hercules CA.* (ed.) B.-R. Laboratories. (Bulletin).

Spjotvoll, T.S.E. (1982). Plots of P-values to evaluate many tests simultaneously. *Biometrika* 69(3)**,** 493-502.

Steinmetz, L.M., Sinha, H., Richards, D.R., Spiegelman, J.I., Oefner, P.J., McCusker, J.H., et al. (2002). Dissecting the architecture of a quantitative trait locus in yeast. *Nature* 416(6878)**,** 326-330. doi: 10.1038/416326a.

Young, S.G., and Bowman, A.W. (1995). Non-Parametric Analysis of Covariance. *Biometrics* 51(3)**,** 920-931. doi: 10.2307/2532993.
